# Apparent PCE Adsorption Characteristics and Low Water-to-Binder Ratio Performance Response of UHPC with Carbonate Manufactured Sand

**DOI:** 10.3390/ma19214517

**Published:** 2026-10-27

**Authors:** Qinfan Bao, Yuqiu Wang, Baoliang Man, Yonghui Han, Wencong Zhang, Xinlin Wang, Rongqing Qi, Rongxin Guo

**Affiliations:** 1 Yunnan Yunling Bridge Intelligent Construction Co., Ltd., Kunming 650200, China; 2 Yunnan Key Laboratory of Disaster Reduction in Civil Engineering, Faculty of Civil Engineering and Mechanics, Kunming University of Science and Technology, Kunming 650500, Chinaqrqing@swfu.edu.cn (R.Q.); 3 Yunnan International Joint Laboratory of Green Construction and Intelligent Maintenance, Faculty of Civil Engineering and Mechanics, Kunming University of Science and Technology, Kunming 650500, China; 4 College of Civil Engineering, Southwest Forestry University, Kunming 650224, China

**Keywords:** ultra-high performance concrete (UHPC), manufactured sand, dolomite, polycarboxylate ether superplasticizer (PCE), adsorption, fresh rheological properties

## Abstract

Carbonate manufactured sand (crushed sand from natural rock) offers an alternative to river and quartz sands in ultra-high-performance concrete (UHPC). This study compared calcite–dolomite sand with calcite-dominated sand. X-ray diffraction and fluorescence characterized mineralogical and chemical compositions. Total organic carbon analysis assessed apparent adsorption of a polycarboxylate ether superplasticizer (PCE). Flowability, fresh rheological properties, strengths, and cross-sectional visible porosity were evaluated at water-to-binder (w/b) ratios of 0.17, 0.16, and 0.15. The calcite–dolomite sand showed a 2.23% higher gradation-weighted apparent PCE adsorption index than the calcite-dominated sand. At w/b = 0.15, UHPC containing calcite–dolomite sand had a 6.45% lower flow spread, a 61.7% longer T500, a 17.5% higher dynamic yield stress, a 21.5% higher plastic viscosity, and a 7.0% higher visible pore area fraction than UHPC containing calcite-dominated sand. At matched w/b ratios, UHPC containing calcite–dolomite sand had 2.3–2.9% lower compressive strength than UHPC containing calcite-dominated sand; the flexural strength deficit increased from 8.0% to 16.0% as w/b decreased. Greater apparent PCE adsorption by sand may reduce dispersant availability, weaken particle dispersion, and impede air release through increased viscous resistance at low w/b ratios. A source-dependent effective PCE dosage window is proposed as a conceptual framework.

## 1. Introduction

Ultra-high performance concrete (UHPC) has broad application prospects in major infrastructure, marine engineering, and special protective engineering, owing to its excellent mechanical properties, durability, and potential for structural lightweighting [1,2]. UHPC is typically proportioned with an ultra-low water-to-binder (w/b) ratio and a high cementitious materials content to achieve a dense microstructure and ultra-high strength [3,4,5]; however, this also renders the fresh paste highly sensitive, characterized by a high solid volume fraction and a low free water content, and makes the workability of the mixture particularly susceptible to variations in the effective dosage of chemical admixtures and in raw material composition [6,7]. As the solid constituent occupying the largest volume fraction in UHPC, fine aggregate is subject to fluctuations in source and properties, which are often amplified and reflected as instability in construction performance and variations in strength [8,9,10].

Traditional UHPC generally uses river sand or quartz sand as fine aggregate. However, owing to constraints on river sand resources and requirements for ecological environmental protection, river sand mining is becoming increasingly restricted [11,12,13,14]; the mining, processing, and use of quartz sand are also accompanied by high energy consumption and the risk of exposure to crystalline silica dust [15,16,17]. Manufactured sand has advantages such as wide raw material availability, controllable production scale, and relatively low engineering cost, and has therefore become an important alternative fine aggregate for UHPC [18]. In this study, manufactured sand refers specifically to crushed sand obtained by mechanically crushing and screening natural carbonate rock [19,20]. The term describes the processing route of the sand, while its mineral constituents originate from natural rock. The investigated materials are therefore distinguished from industrial by-product aggregates and specially produced artificial aggregates. Both these sands and river sand originate from natural mineral resources. Quartz sand produced by crushing quartz-rich rock can also be classified as manufactured or crushed sand; the distinction between quartz and carbonate sands concerns their mineral composition. The present work focuses on two carbonate manufactured sands with different mineralogical characteristics. Compared with river sand and industrial quartz sand, the particle morphology, fine particle content, and mineral composition of manufactured sand are significantly influenced by the lithology of the parent rock and the crushing process [21], and manufactured sands from different sources often exhibit simultaneous differences in multiple properties [11,22]. In particular, for carbonate manufactured sand, the parent rock may contain both calcite (CaCO_3_) and dolomite (CaMg(CO_3_)_2_). Previous studies have shown that the rheological effect of manufactured-sand microfines depends not only on their content, but also on their mineral type, particle shape, packing characteristics, frictional contacts, and polycarboxylate ether superplasticizer (PCE) surface coverage [23]. Differences in mineral composition and surface characteristics resulting from different sand sources may further affect the compatibility of the sand with the cementitious materials and chemical admixture system of UHPC.

Existing studies have mainly examined the effects of manufactured sand on the properties of cement-based materials from the perspectives of replacement ratio, stone powder content, particle gradation, particle morphology, and clay impurities [11,12,18,22,24]. The relevant results indicate that an appropriate amount of fine particles can improve particle packing and the hardened matrix structure through a filling effect, whereas excessively high fine powder content, poor particle morphology, or clay impurities may increase the water demand and PCE consumption of the system and reduce the workability of the mixture. Meanwhile, the adsorption and distribution of PCE on the surfaces of cement, silica fume, and mineral particles are important factors affecting the dispersion and rheological behavior of pastes with low w/b [6,7,25]. However, the mineral composition, particle morphology, pore characteristics, and surface properties of manufactured sand in actual engineering are usually coupled, and it is difficult to simply attribute their effects to a single mineral [26,27,28]. Compared with studies on the stone powder content or replacement ratio of manufactured sand, systematic analysis is still lacking regarding the differences in multi-indicator responses of carbonate manufactured sands from different engineering sources across low w/b ratios. In particular, evaluating the compatibility of manufactured sand solely by the final spread diameter or compressive strength may not fully reflect the changes in dynamic flow resistance, plastic viscosity, and flexural performance with the presence of fibers.

This study investigated two carbonate manufactured sands: a calcite–dolomite sand (S1) and a calcite-dominated sand (S2). After sieving, the two manufactured sands were adjusted to the same mass gradation. With the cementitious material composition, sand content, steel fiber dosage, and PCE dosage kept consistent, three w/b ratios of 0.17, 0.16, and 0.15 were adopted. Total organic carbon (TOC) analysis was used to characterize the apparent PCE consumption of different size fractions of the manufactured sands. The flow spread, T500, dynamic yield stress, and plastic viscosity of fresh UHPC mixtures, as well as the compressive and flexural strengths after hardening, were tested. The relative responses of the measured properties were compared to assess how the sand-source differences varied with decreasing w/b and to identify the most sensitive performance indicators. The findings can provide a reference for raw material screening, workability evaluation, and mix proportion compatibility of carbonate manufactured sand in UHPC with ultra-low w/b ratios.

## 2. Materials and Methods

### 2.1. Raw Materials

UHPC specimens were prepared with the two manufactured sands used in this study. The materials used included: Huaxin P.O. 52.5 ordinary Portland cement (OPC) (Kunming, China); semi-densified silica fume (SF) from Fangke New Material Technology Co., Ltd. (Xi’an, China), with a SiO_2_ content of 96.54%; Class II fly ash (FA) from Xuanwei Power Plant (Xuanwei, China), with a 28-day activity index of 81%; polycarboxylate superplasticizer (PCE) from Sobute New Materials Co (Nanjing, China).; and copper-coated straight steel fiber (Fiber) with a length of 13 mm, a diameter of 0.2 mm, and an aspect ratio (l/d) of 65.

**Figure 1:**
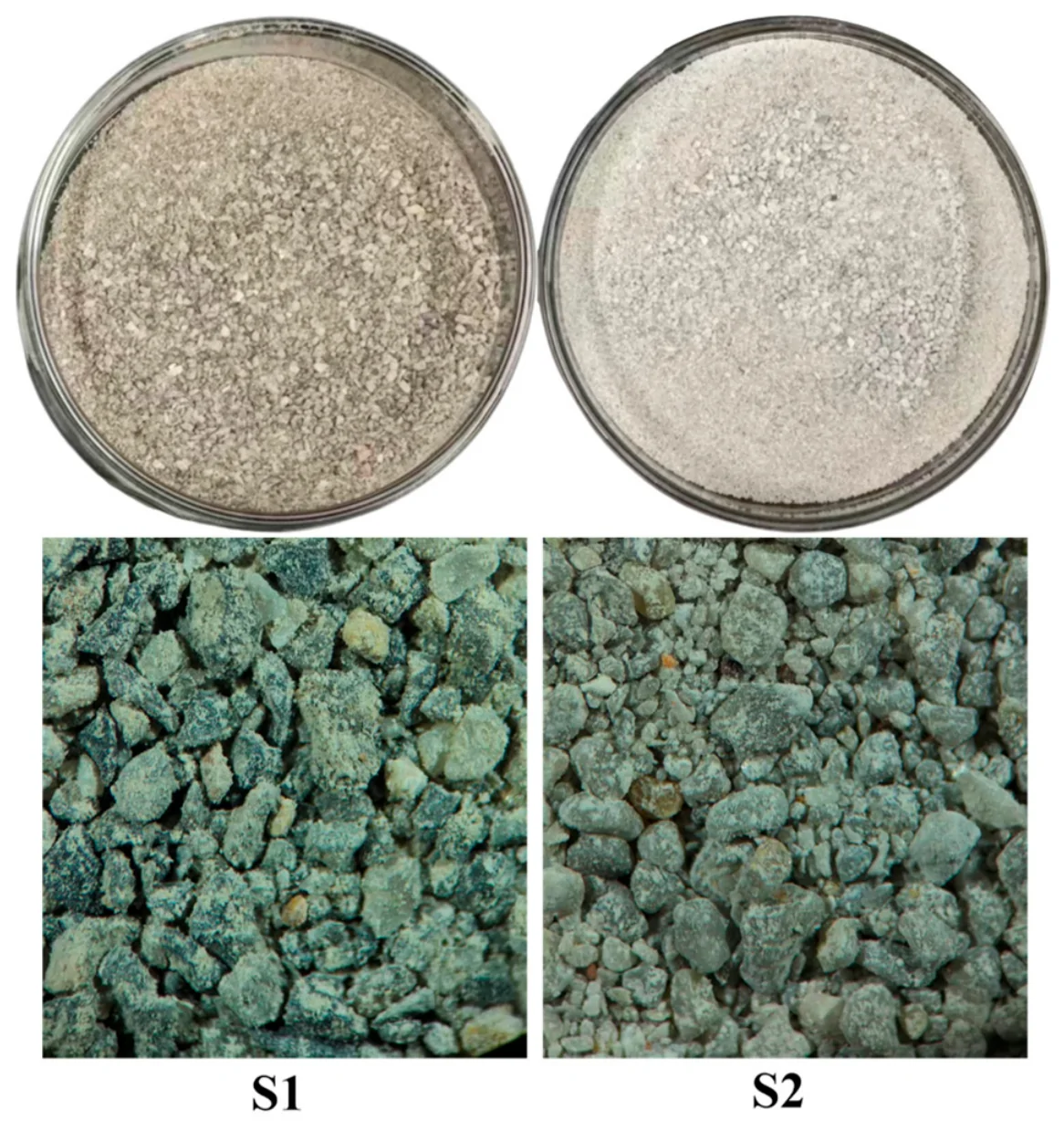
Photographs and microscopic images of the two manufactured sands.

**Figure 2:**
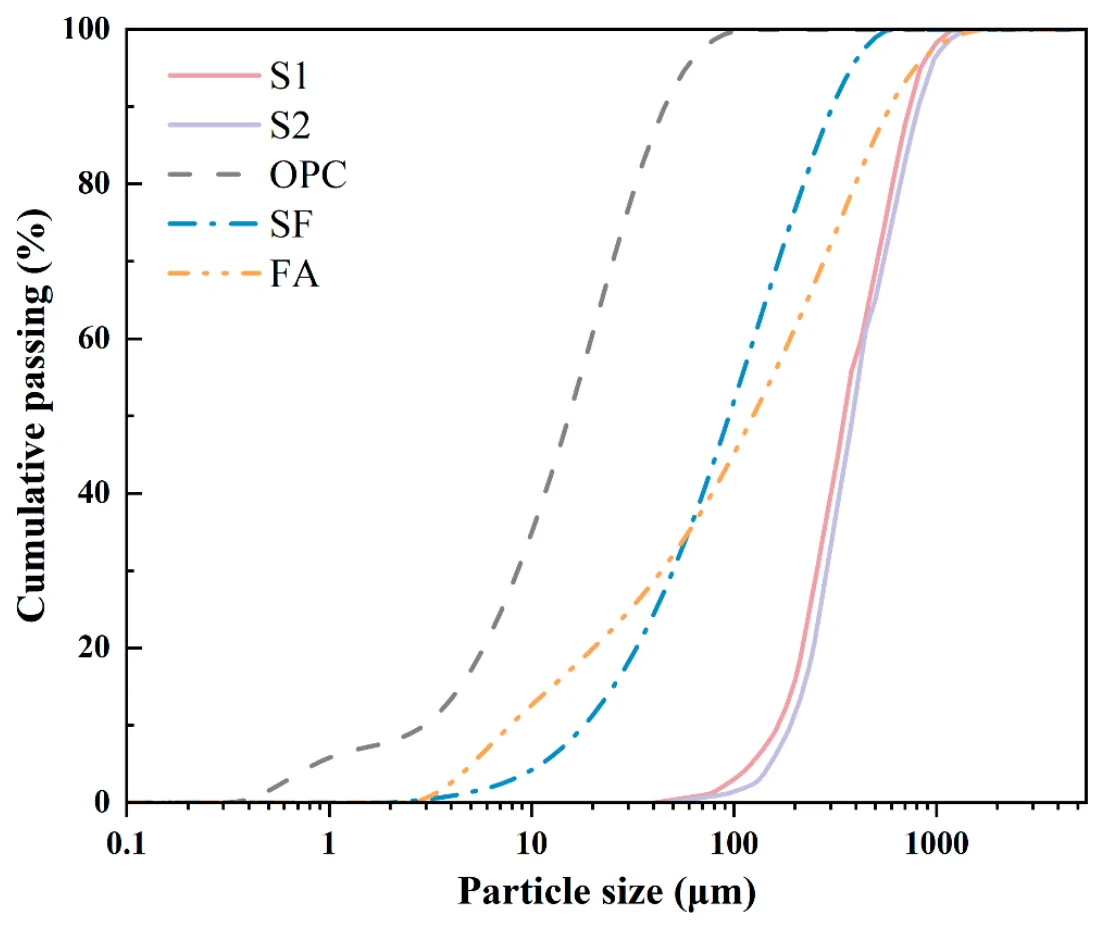
Cumulative particle size distributions of the raw materials.

Two carbonate manufactured sands from different engineering sources were used as fine aggregates, denoted as S1 and S2. S1 was manufactured sand from Yuzhou Stone Plant Yunlong County (Dali, China), and S2 was manufactured sand from Yunnan Hongyan Zhanfeng Sand Field (Dali, China). Both manufactured sands were air-dried prior to testing. The micrographs are shown in Figure 1 and the particle size distribution curves of the various materials are shown in Figure 2. For the XRF analysis, the manufactured sand samples were ground and prepared as pressed powder pellets. The analytical results were requested in oxide-equivalent forms, and the oxide-equivalent results are presented in Table 1. A separate loss-on-ignition (LOI) determination was not requested. The basic properties of the two manufactured sands are listed in Table 2.

**Figure 3:**
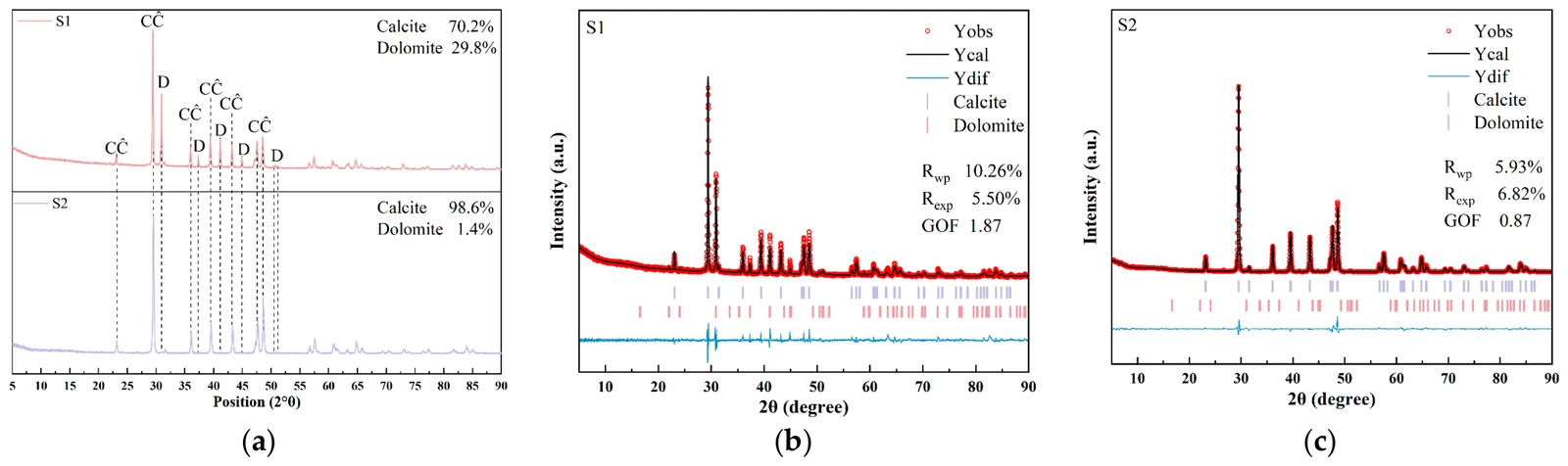
XRD characterization of the two manufactured sands: (**a**) phase identification; (**b**,**c**) Rietveld refinement results (CĈ = Calcite, D = Dolomite).

Representative samples from the entire batch of manufactured sand were ground for mineral composition analysis. Figure 3 shows the XRD patterns of the two manufactured sands. The phase analysis results indicate that among the detected crystalline minerals, S1 is mainly composed of calcite and dolomite, whereas S2 is dominated by calcite. The XRF results in Table 1 show that the MgO contents of S1 and S2 are 6.08% and 0.83%, respectively, which are basically consistent with the mineral composition differences reflected by XRD. Therefore, S1 is hereafter referred to as calcite–dolomite manufactured sand, and S2 as calcite-dominated manufactured sand.

### 2.2. Mix Proportions

After sieving at the original production facility, the two sands were divided into the following four size fractions: 0.6–1.18 mm, 0.3–0.6 mm, 0.15–0.3 mm, and 0.075–0.15 mm. Each fraction was used in a mass ratio of 1:1:1:1. In preparing UHPC specimens, the two manufactured sands were used separately with different w/b ratios, and the mix proportions are shown in Table 3.

The w/b was calculated by dividing the combined mass of the separately added mixing water and the water contained in the PCE solution by the total mass of OPC, SF, and FA. In Table 3, “PCE” denotes the mass of the commercial superplasticizer solution. The PCE solution had a solid content of 50% by mass; therefore, its dosage of 24 kg/m^3^ introduced 12 kg/m^3^ of water, which was included in the free-water content used for the w/b calculation. The total binder content was 1150 kg/m^3^, comprising 800 kg/m^3^ OPC, 180 kg/m^3^ SF, and 170 kg/m^3^ FA. Accordingly, the separately added water contents of 183, 172, and 160 kg/m^3^ resulted in calculated w/b values of 0.17, 0.16, and 0.15.

Throughout this study, S1 and S2 denote the two manufactured sands, whereas the S1 group and S2 group refer to the UHPC mixtures prepared with S1 and S2, respectively. Individual mixtures are designated by the sand type followed by the nominal w/b ratio; for example, S1-0.15 denotes the UHPC mixture prepared with S1 at a nominal w/b ratio of 0.15.

### 2.3. UHPC Preparation and Curing

**Figure 4:**
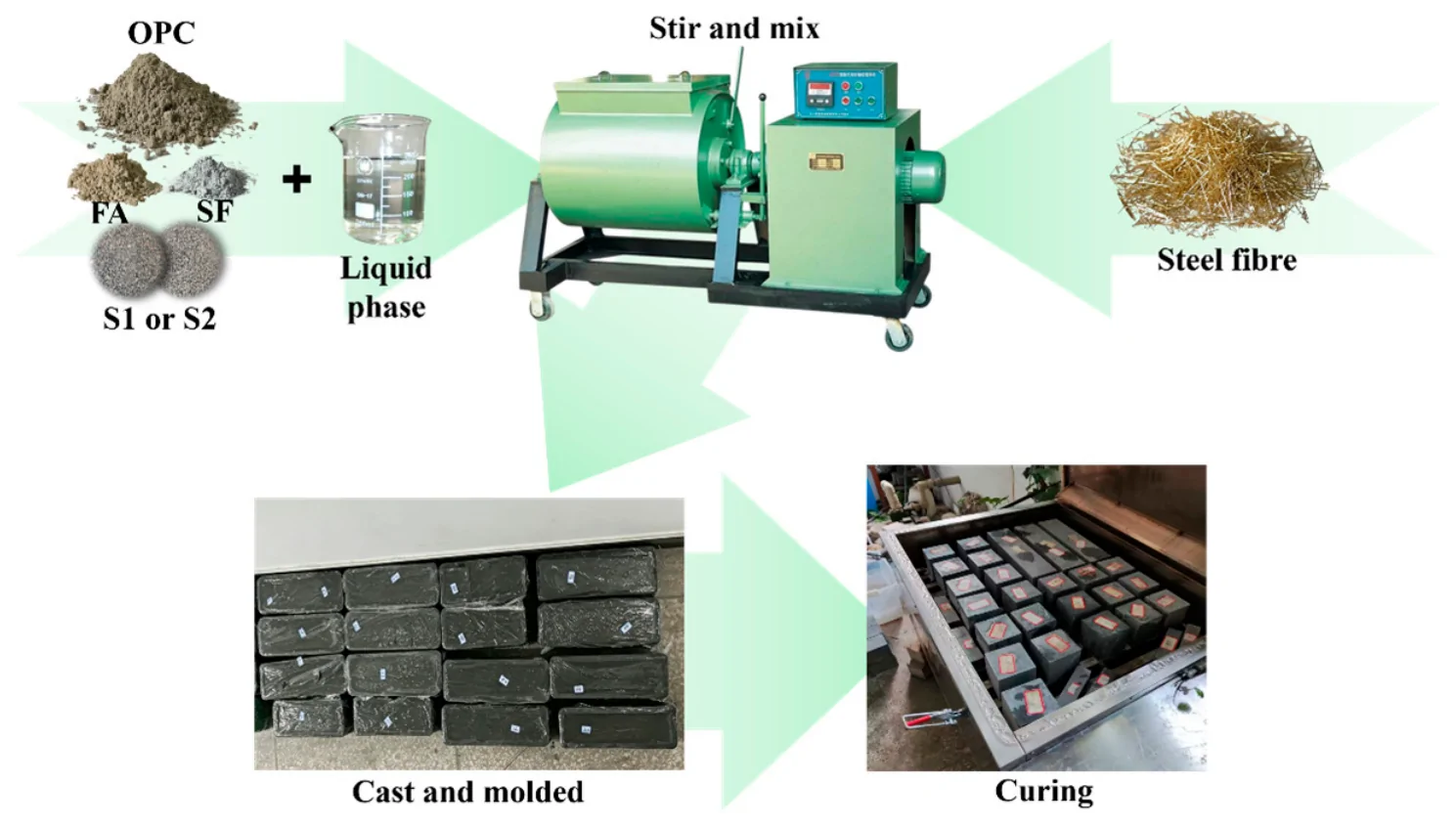
UHPC specimen preparation procedure.

The preparation procedure used in this study was as follows. The raw materials were weighed and added into a single-shaft horizontal forced-action mixer (HJW-60). The mixer had a maximum feed capacity of 60 L, and the batch volume used in this study was 20 L. The materials were dry pre-mixed for 5 min. PCE and water were weighed separately and thoroughly mixed. The mixed solution was then added into the mixer in two portions, with an interval of 120 s between the first and second additions. After the second addition, mixing was continued for another 300 s. Steel fibers were uniformly sprinkled manually into the mixer within a controlled period of 120 s. After all fibers had been added, mixing was continued for a further 200 s before discharge. The flow chart of the specimen preparation process is shown in Figure 4.

**Figure 5:**
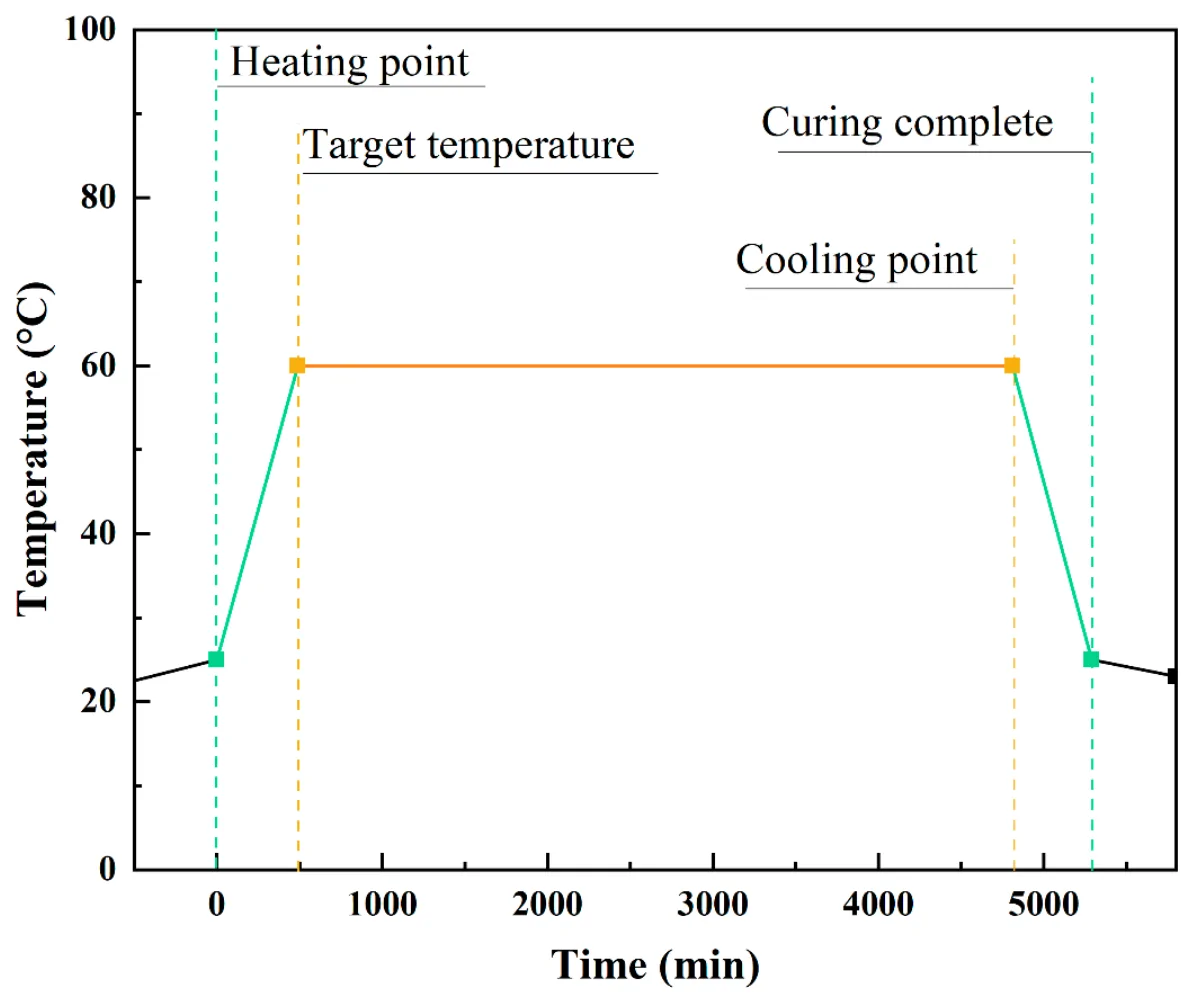
Steam-curing procedure.

After discharge, the fresh mixture was poured into six 100 mm × 100 mm × 100 mm cube molds and three 100 mm × 100 mm × 400 mm prism molds for compressive and flexural strength tests, respectively. After casting, the specimens were covered with polyethylene film and kept in a curing room at 20 ± 1 °C and 99% relative humidity for 24 h. They were then transferred to a steam-curing chamber, heated to 60 °C, and maintained at this temperature for 72 h. After cooling to room temperature inside the chamber, the specimens were stored under laboratory conditions (23 ± 2 °C and 50% relative humidity) and tested at a total age of 5 days. Figure 5 shows the steam curing scheme used in this research.

### 2.4. TOC-Based Apparent PCE Adsorption Test

TOC analysis has been widely used to determine residual PCE-related organic carbon in the liquid phase and characterize relative adsorption behavior [29]. Here, the TOC concentrations of the PCE solution were measured before and after contact with each sand under the same aqueous test conditions. The concentration difference was used to calculate an apparent PCE adsorption index.

**Figure 6:**
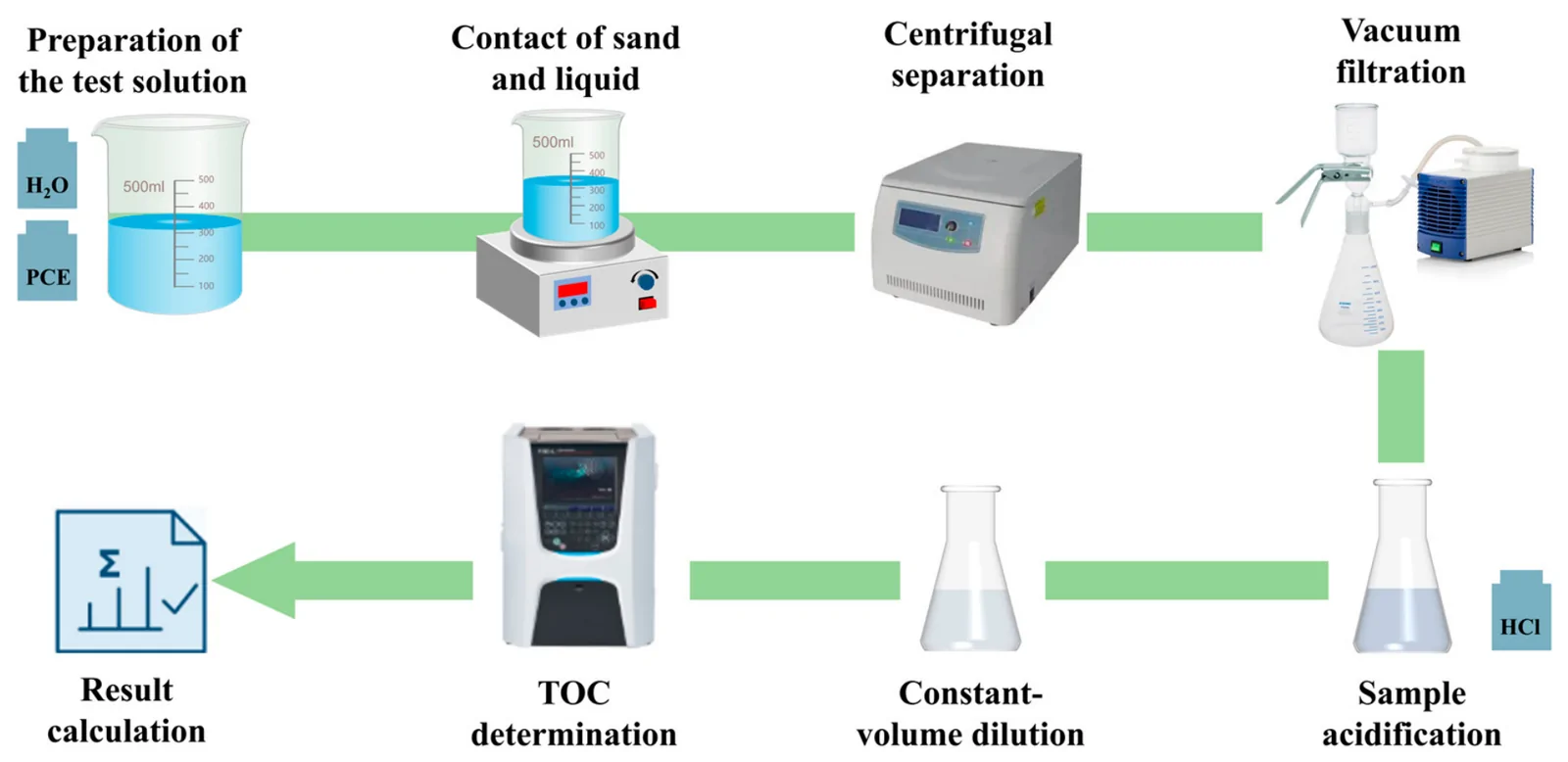
Procedure for the TOC-based apparent PCE adsorption test.

The test procedure is shown in Figure 6, and the detailed steps are as follows. A total of 400 g of deionized water and 50 g of PCE were weighed and thoroughly mixed to prepare the test solution. A 15.0 g sand sample was weighed and mixed with 22.5 g of the test solution. After magnetic stirring for 10 min, the mixture was left to stand for 2 min. The upper liquid phase was collected and centrifuged at 4000 r/min for 10 min. The supernatant was then passed through a nylon microporous membrane with a pore size of 0.22 μm. A 60 mg aliquot of the filtrate was weighed, acidified with 120 mg of 1 mol/L hydrochloric acid, and diluted 800-fold with deionized water. The TOC concentration of the diluted solution was measured using a vario TOC select total organic carbon analyzer (Elementar, Langenselbold, Germany). The apparent PCE adsorption index was calculated using Equation (1). Three separately prepared samples were tested for each condition, and the results are reported as mean values.

(1) $$T=\frac{(C_{0}-C_{1})\times V}{m}$$

where *T* is the apparent organic carbon consumption per unit mass of manufactured sand (mg C/g); *C*_0_ and *C*_1_ are the PCE-related organic carbon concentrations in the liquid phase before and after contact with the sand, respectively (mg C/L); *V* is the volume (L); and *m* is the mass of the sand sample (g). It should be noted that the calculated PCE adsorption index can be used as a comparison indicator to assess the degree of organic carbon loss related to PCE when two sands come into contact under the same test conditions.

### 2.5. Flowability Tests

The flowability test in this study was conducted using a base plate measuring 1500 mm × 1500 mm with a thickness of 5 mm, and a slump flow cone with a top diameter d of 100 mm, a bottom diameter D of 200 mm, and a height h of 300 mm. The UHPC mixture was poured into the cone, after which the cone was slowly lifted vertically to allow the mixture to flow freely from the bottom. Timing was started simultaneously to record the T500. When the mixture reached a circular outline with a diameter of 500 mm, the elapsed time was recorded as the flow time to 500 mm spread. When the total time reached 90 s, two mutually perpendicular diameters were measured, and the arithmetic mean of the two values was taken as the flow spread of that batch of UHPC. Flow spread and T500 were measured on two separately collected samples from the same UHPC batch, and the reported values are their means.

### 2.6. Fresh Rheological Test

**Figure 7:**
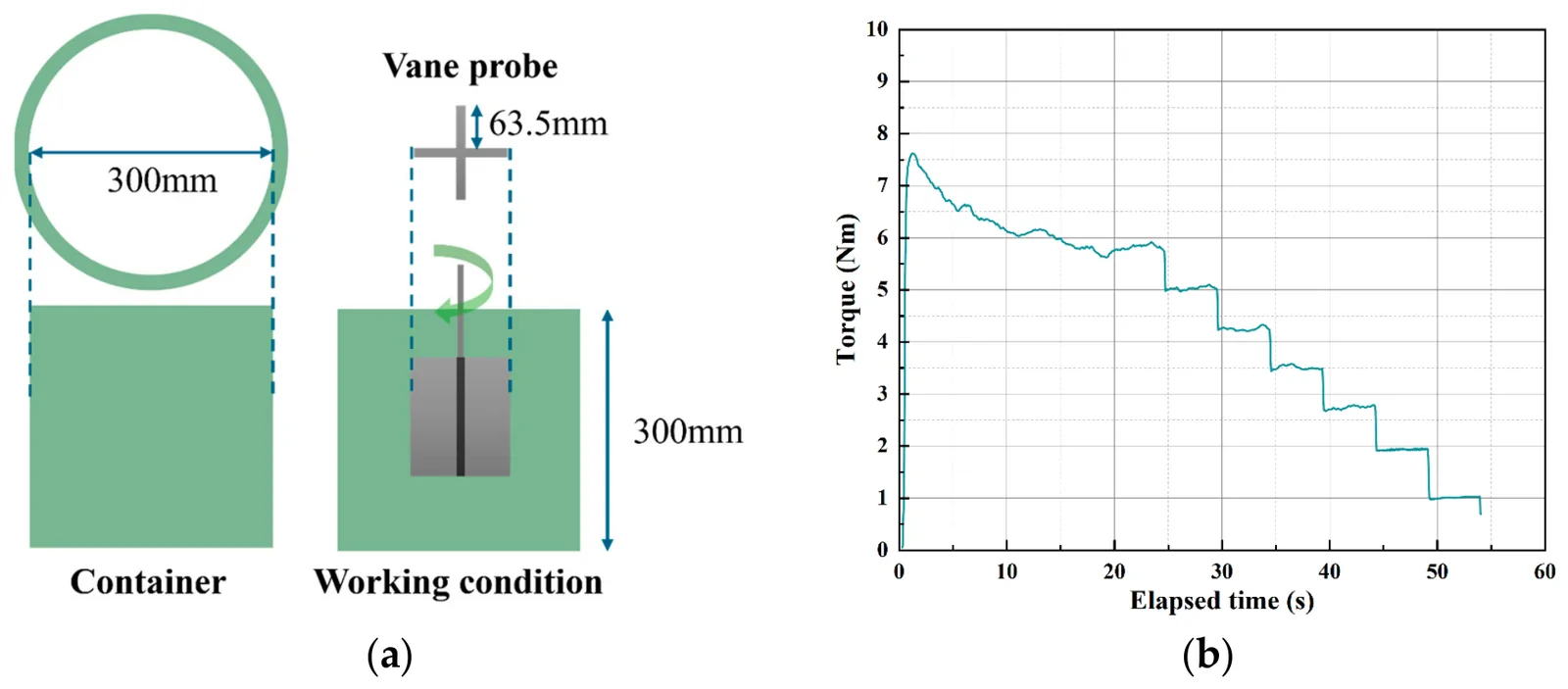
(**a**) Vane and container dimensions; (**b**) fresh rheological test setup.

Fresh rheological properties were measured using an ICAR rheometer, which provides dynamic yield stress and plastic viscosity from flow-curve measurements [30,31,32]. The geometry inputs were a vane radius of 63.5 mm, a vane height of 127.0 mm, and an effective container radius of 143.0 mm. To minimize the interference of early structural build-up and time effects after mixing on the results, pre-shearing was performed at a rotational speed of 0.500 rev/s for 20 s. Subsequently, a descending rotational-speed test was conducted, in which the rotational speed was decreased stepwise from 0.500 to 0.050 rev/s in seven stages, each lasting 5 s. The rheological parameters were determined by fitting the descending segment of the torque–rotational speed curve with the Bingham model. The rheological test procedure was as follows: after mixing, the fresh mixture was taken out and placed into the test container; the rheometer was positioned, the vane was inserted into the mixture, and the instrument was locked; and testing was started after the mixture gradually returned to a stable state. The vane and container dimensions and the rheological test setup are shown in Figure 7. For each mixture, the rheological parameters were determined in duplicate using separately collected samples from the same batch of freshly mixed UHPC.

The Bingham model relates shear stress to shear rate as Equation (2):

(2) $$\tau =\tau_{0}+\mu_{p}\dot{\gamma }$$

where *τ* is the shear stress (Pa), *τ*_0_ is the dynamic yield stress (Pa), *μ*_p_ is the plastic viscosity (Pa·s), and $\dot{\gamma }$ is the shear rate (s^−1^).

The conversion of torque–rotational speed data is based on the Reiner–Riwlin formulation. The vane is represented by an equivalent cylinder, and torque contributions from its ends are neglected. With the angular velocity defined as (Ω = 2*πN*), the relationship is given by Equation (3):

(3) $$\Omega =\frac{T}{4\mathrm{\pi h}\mu_{p}}\left(\frac{1}{R_{i}^{2}}-\frac{1}{R_{e}^{2}}\right)-\frac{\tau_{0}}{\mu_{p}}\ln\left(\frac{R_{e}}{R_{i}}\right)$$

The effective outer radius of the yielded region is given by Equation (4):

(4) $$R_{e}=\min\left[R_{o},\sqrt{\frac{T}{2\mathrm{\pi h}\tau_{0}}}\right]$$

Here, *T* is the measured torque (N·m), *N* is the rotational speed (rev/s), Ω is the angular velocity (rad/s), *R*_i_ is the vane radius, *R*_o_ is the effective container radius, and *h* is the vane height. The geometry inputs were *R*_i_ = 0.0635 m, *R*_o_ = 0.1430 m, and *h* = 0.1270 m. When the entire annular gap is yielded, (*R*_e_ = *R*_o_); otherwise, (*R*_e_ < *R*_o_), accounting for an unyielded region near the outer boundary. The software determines *τ*_0_ and *μ*_p_ from the measured flow-curve data using these relationships, applying a nonlinear solution when partial yielding occurs.

### 2.7. Mechanical Properties Test

The mechanical properties of the UHPC specimens were tested using a WANCE HCT206E microcomputer-controlled electro-hydraulic servo testing machine (WANCE, Shenzhen, China). The maximum load capacity was 2000 kN, and the loading frame was a four-column hydraulic frame with high rigidity. The sensitivity of the load sensor was 2.3 mV/V. In the compressive strength test, a preload of 500 N was applied, and the loading rate was maintained at 2.5 kN/s; the reported compressive strength was the average of six specimens. In the flexural strength test, four-point bending was adopted, with a support span of 300 mm and a distance of 100 mm between the two loading points. A preload of 200 N was applied, and the loading rate was maintained at 333 N/s; the reported flexural strength was the average of three specimens.

### 2.8. Pore Image Analysis

For each UHPC mixture, the left and right end sections of the same prismatic specimen were selected for pore analysis. The section surfaces were photographed under high-contrast illumination, under which the visible pores appeared dark and could be distinguished from the gray cementitious matrix. The same image-acquisition settings were applied to all mixtures, and each image was cropped to the effective cross-sectional region with a size of 1350 × 1279 pixels and a spatial resolution of 0.0782 mm/pixel.

The acquired images were processed using Fiji. Each image was converted to 8-bit grayscale, followed by smoothing and filtering to suppress local grayscale fluctuations and image noise. Threshold segmentation was then performed to separate the dark pore regions from the surrounding matrix, producing binary images in which the identified pores were represented as foreground objects. This procedure followed the general principles of automated two-dimensional void analysis for contrast-enhanced cementitious sections [33,34].

The binary images were subsequently imported into MATLAB (9.14.0.2206163 (R2023a)) for quantitative analysis. Individual pores were identified as independent eight-connected regions, and their actual projected areas, centroids, and equivalent diameters were calculated.

## 3. Results

### 3.1. Apparent PCE Adsorption Differences

**Figure 8:**
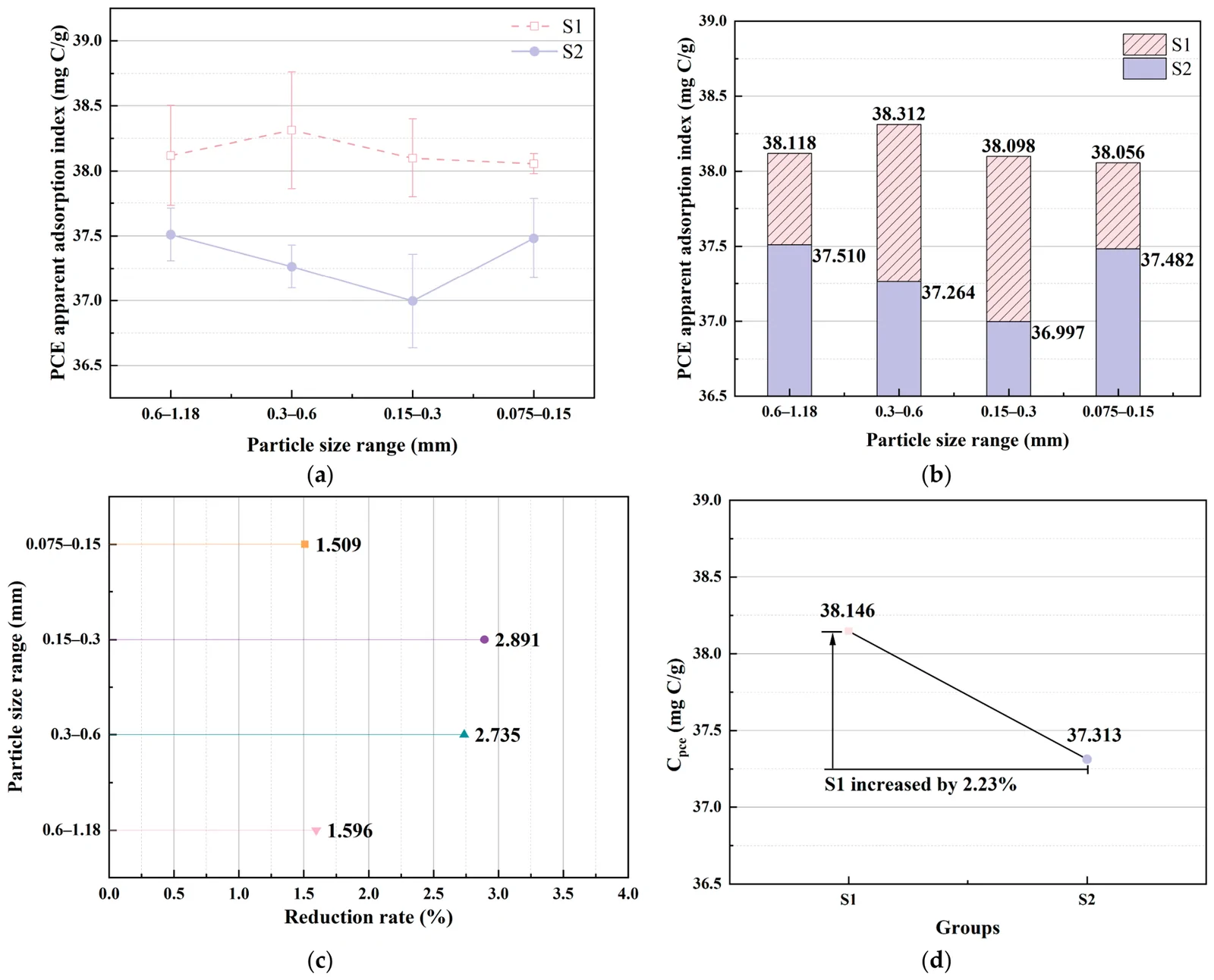
Comparison of PCE adsorption between two types of manufactured sand. (**a**) adsorption trends of PCE at different particle sizes; (**b**) adsorption indicators of PCE for different particle sizes; (**c**) the decrease rate of adsorption of S2 relative to S1; (**d**) the difference in weighted apparent PCE adsorption index between the two types of sands. Values in (**a**,**b**) are means of three separately prepared sand–solution test samples for each sand type and size fraction (*n* = 3). Error bars in (**a**) represent the sample standard deviation (SD). The indices in (**c**,**d**) were calculated from the reported mean values.

Figure 8 compares the TOC-derived apparent PCE adsorption indices of the two sands across the tested size fractions. A higher value indicates a stronger adsorption or solid-phase retention effect of the manufactured sand on PCE-related organic components.

As shown in Figure 8a,b, within the four size fractions of 0.6–1.18 mm, 0.3–0.6 mm, 0.15–0.3 mm, and 0.075–0.15 mm, the apparent PCE adsorption values of S1 are all higher than those of S2. The values for S1 at these size fractions are 38.118, 38.312, 38.098, and 38.056 mg C/g, respectively, ranging from 38.056 to 38.312 mg C/g; the corresponding values for S2 are 37.510, 37.264, 36.997, and 37.482 mg C/g, respectively, ranging from 36.997 to 37.510 mg C/g. The absolute differences between the two sands at the same size fractions are 0.608, 1.048, 1.101, and 0.574 mg C/g, respectively. Across all four compared size fractions, S1 showed higher mean apparent PCE adsorption indices than S2.

To further compare the adsorption differences between the two manufactured sands at different size fractions, the reduction rate of the apparent PCE adsorption value of S2 relative to S1 was calculated, and the results are shown in Figure 8c. At the size fractions of 0.6–1.18 mm, 0.3–0.6 mm, 0.15–0.3 mm, and 0.075–0.15 mm, the values for S2 are lower than those for S1 by 1.596%, 2.735%, 2.891%, and 1.509%, respectively. The largest difference occurs at the 0.15–0.3 mm fraction, followed by the 0.3–0.6 mm fraction, while the difference at the 0.075–0.15 mm fraction is relatively small. These results indicate that the difference in PCE adsorption between the two manufactured sands does not exhibit a simple monotonic variation with decreasing particle size. Calculated from the absolute differences at each size fraction, the two intermediate fractions of 0.3–0.6 mm and 0.15–0.3 mm together account for approximately 64.5% of the total difference across all fractions, suggesting that the intermediate size fractions may be an important source of the PCE adsorption difference between the two blended sands. Therefore, the adsorption difference between the two sands cannot be explained solely by the decrease in particle size and the increase in specific surface area, but may also be influenced by the combined effects of mineral composition, particle morphology, and surface state of each size fraction.

Since the UHPC specimens used manufactured sand blended from different size fractions, to relate the single-fraction test results to the actual sand condition, a gradation-weighted calculation was further performed according to the mass ratio of 1:1:1:1 for the four size fractions of 0.6–1.18 mm, 0.3–0.6 mm, 0.15–0.3 mm, and 0.075–0.15 mm. The gradation-weighted index was calculated using Equation (5):

(5) $$C_{\mathrm{PCE}}=\sum_{i=1}^{4}w_{i}C_{i}$$

where *C*_PCE_ denotes the gradation-weighted apparent PCE adsorption index, *C_i_* is the index for size fraction *i*, and *w_i_* is its mass fraction in the blended sand. The four fractions were blended in equal mass proportions, giving *w_i_* = 0.25.

Figure 8d shows gradation-weighted apparent PCE adsorption indices of 38.146 mg C/g for S1 and 37.313 mg C/g for S2. The index for S1 was 2.23% higher than that for S2, consistent with its higher mean indices across all four size fractions.

Combined with the aforementioned XRD and chemical composition analyses, the presence of dolomite components in S1 may be one of the reasons for the difference in PCE adsorption between the two manufactured sands. Dolomite and calcite differ in crystal structure, surface cation composition, and the types of potential active sites. The ordered distribution of Ca^2+^ and Mg^2+^ in the dolomite crystal lattice may alter the local surface charge and ionic environment of the particles, thereby affecting the interaction between the carboxyl groups of PCE molecules and the surface of carbonate particles [24,35,36,37,38]. In addition, the fresh fracture surfaces, structurally disordered regions, and surface defects generated during the crushing and processing of manufactured sand may also increase the potential adsorption sites for PCE molecules [39]. The higher apparent PCE adsorption index of S1 may reflect the combined influence of its mineral composition and particle surface characteristics.

### 3.2. Flow Spread and T500

**Figure 9:**
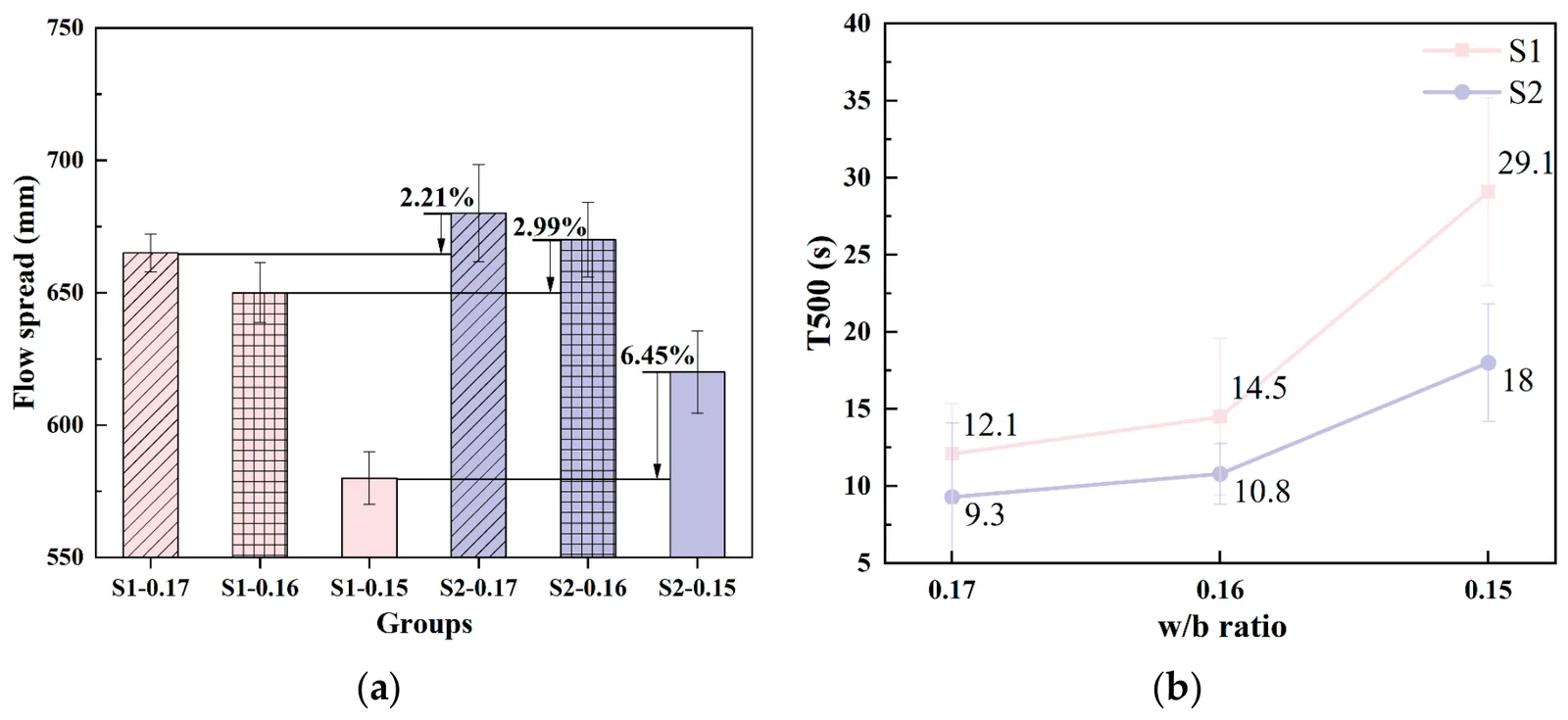
The working performance of the UHPC slurry in the two groups. (**a**) flow spread; (**b**) T500. Values are means of test results from two separately collected samples from a single UHPC batch (*n* = 2 samples; one batch). Error bars represent the sample SD across the two sample-level results and describe within-batch sampling and measurement variation.

Figure 9 compares the flow spread and T500 of the two UHPC groups. As the w/b ratio decreased from 0.17 to 0.15, flow spread decreased and T500 increased in both groups. At each w/b ratio, the S1 group had a smaller flow spread and a longer T500 than the S2 group.

As shown in Figure 9a, at w/b ratios of 0.17, 0.16, and 0.15, the flow spread of the S1 group was 2.21%, 2.99%, and 6.45% lower than that of the S2 group, respectively. At a w/b ratio of 0.17, the difference in flow spread between the two groups was relatively small; however, as the w/b ratio decreased, the gap gradually widened. The relative flow spread deficit of S1 widened as the w/b ratio decreased. At w/b = 0.15, this deficit was approximately 2.92 times that at w/b = 0.17. This result indicates that the flow spread of the S1 group is more sensitive to the decrease in w/b ratio, and that a lower w/b ratio further amplifies the workability difference caused by the two manufactured sands.

The longer T500 values of the S1 group are consistent with its lower flow spread (Figure 9b). At w/b ratios of 0.17, 0.16, and 0.15, the T500 values of the S1 group were 30.11%, 34.26%, and 61.67% longer than those of the S2 group, respectively. With decreasing w/b ratio, the T500 of both groups increased, but the increase was larger in the S1 group. In particular, when the w/b ratio decreased from 0.16 to 0.15, the T500 of the S1 group increased rapidly, indicating that its flow rate is more sensitive to low w/b ratio conditions. Compared with the results at a w/b ratio of 0.17, the relative difference in T500 between the two groups at a w/b ratio of 0.15 expanded to approximately 2.05 times. Compared with the final flow spread, the T500 exhibited a higher sensitivity to changes in flow resistance, suggesting that evaluating the final flow spread alone may not be sufficient to fully reflect the influence of the two manufactured sands on the workability of UHPC.

The gradation-weighted apparent PCE adsorption indices were 38.146 mg C/g for S1 and 37.313 mg C/g for S2, corresponding to a 2.23% higher index for S1 relative to S2. This indicates that after blending according to the actual sand gradation used in UHPC, S1 still exhibits a relatively stronger tendency for PCE adsorption and solid-phase retention. Under the condition that the cementitious material composition, PCE dosage, and sand gradation remain consistent, the stronger PCE adsorption of S1 may reduce the effective PCE available in the liquid phase for dispersing cementitious particles such as cement and silica fume, thereby weakening the electrostatic repulsion and steric hindrance provided by PCE. This makes the particles more prone to agglomeration and the formation of stronger flocculated structures, ultimately manifesting as a reduction in flow spread and a prolongation of T500.

It is noteworthy that the gradation-weighted PCE adsorption difference reflects the inherent material properties of the two manufactured sands, whereas the workability difference between the two UHPC groups continues to widen as the w/b ratio decreases. This indicates that the difference in PCE adsorption between the two manufactured sands does not solely control workability but acts in combination with the free water state in the system. These results reflect the high sensitivity of UHPC with a low w/b ratio to the compatibility between raw materials and PCE [34]. Therefore, the workability changes shown in Figure 9 are consistent in trend with the gradation-weighted PCE adsorption results in Section 3.1: the stronger PCE adsorption tendency of S1 may be one of the factors contributing to the deterioration of its flow performance, and the decrease in w/b ratio further amplifies this raw material compatibility difference.

### 3.3. Fresh Rheological Properties

**Figure 10:**
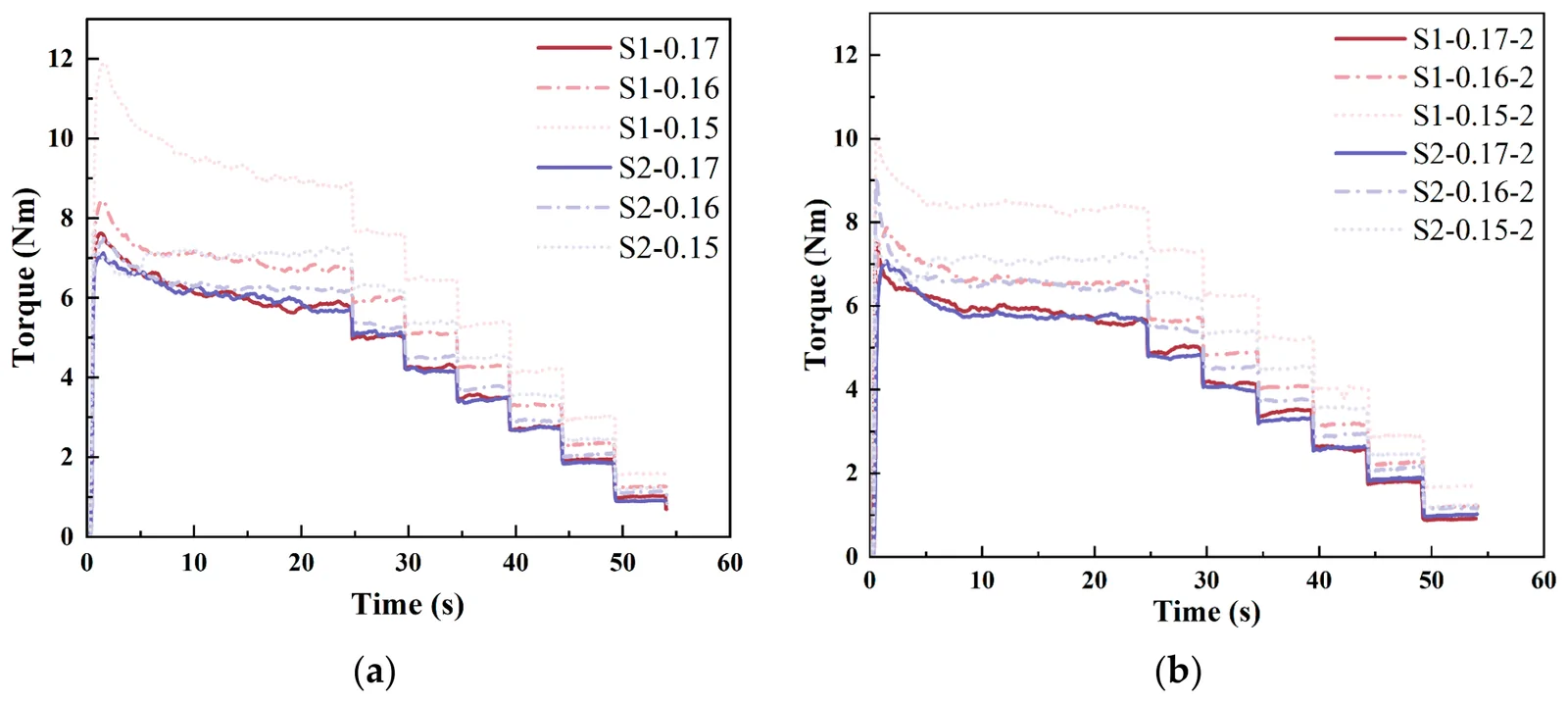
Torque-time responses of each group of slurries. (**a**) first test; (**b**) second test.

Figure 10 shows the torque–time responses during the stepwise reduction in rotational speed. Torque initially decreased during high-speed shearing and then approached a plateau. Each subsequent reduction in rotational speed produced a lower torque plateau.

At a w/b ratio of 0.17, the torque–time curves of S1 and S2 were generally close to each other, suggesting that the difference in shear resistance between the UHPC mixtures prepared with the two manufactured sands was limited when free water was relatively sufficient. When the w/b ratio decreased to 0.16, the two sets of curves began to separate, with the torque of S1 being generally higher than that of S2. When the w/b ratio further decreased to 0.15, the curve for S1 at 0.15 shifted upward significantly and maintained the highest torque level at all rotational speed stages, indicating that the S1 group developed greater flow resistance under low w/b ratio conditions. These changes are consistent with the lower flow spread and longer T500 of S1 reported in Section 3.2.

**Figure 11:**
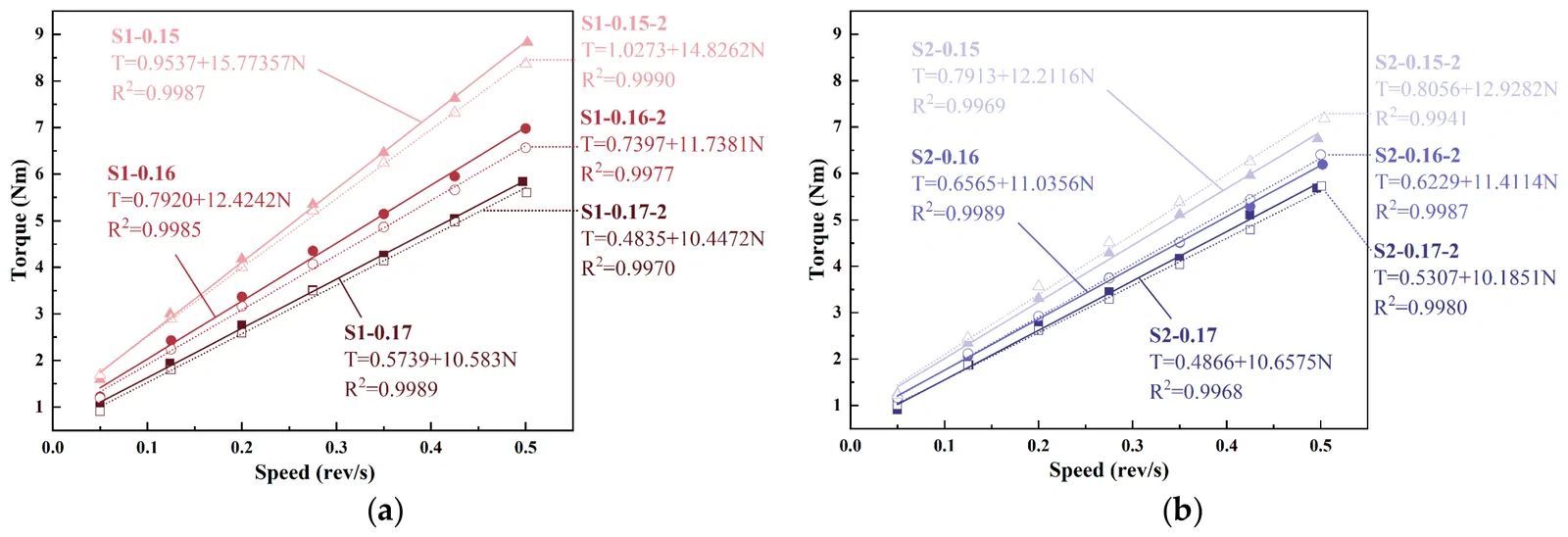

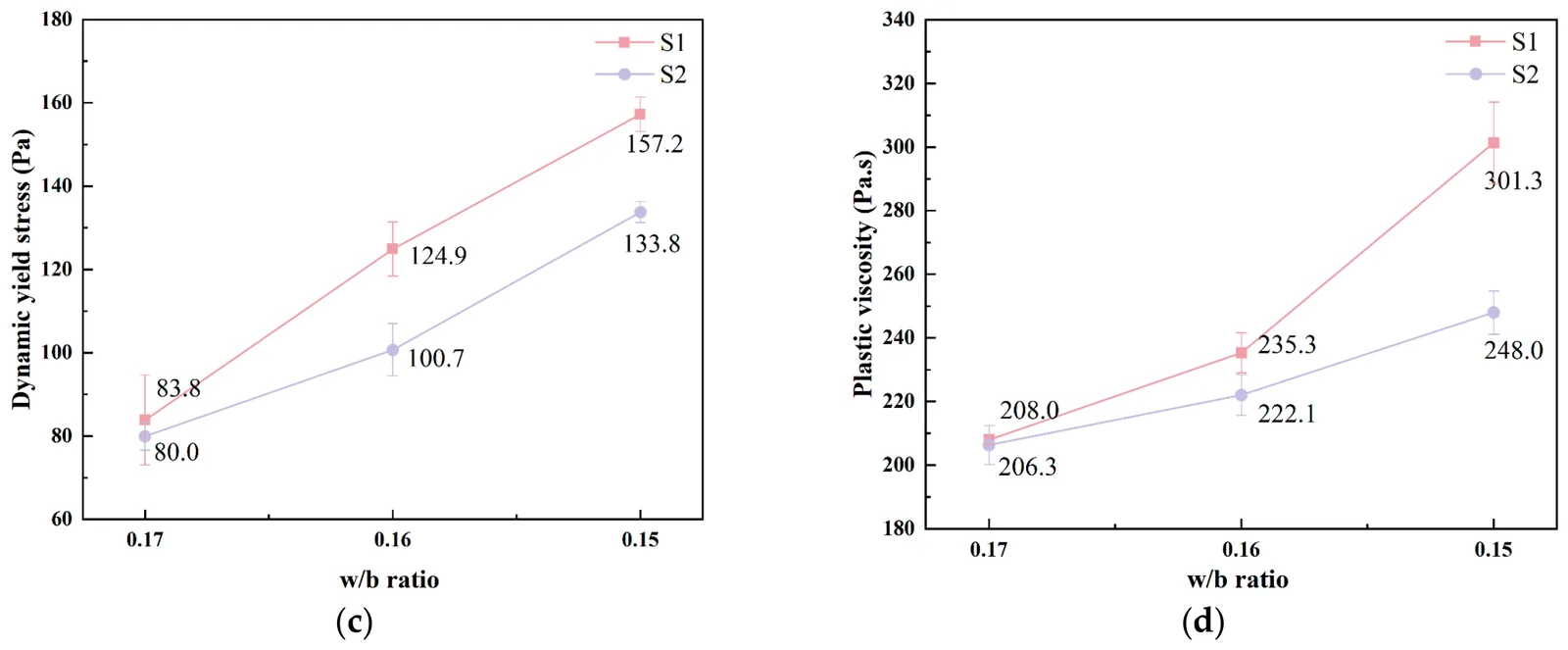
Fresh rheological parameters of each group of slurries. (**a**,**b**) torque versus rotational speed curves; (**c**) dynamic yield stress; (**d**) plastic viscosity. the curves in (**a**,**b**) represent two tests conducted on separately collected samples from a single UHPC batch. The curves in (**a**,**b**) represent two tests conducted on separately collected samples from a single UHPC batch. Values in (**c**,**d**) are the means of these two tests, and error bars represent the sample SD (*n* = 2 samples; one batch). The SD describes within-batch sampling and measurement variation.

Based on the stable torque at each rotational speed stage, the torque–rotational speed relationships were plotted, and the results are shown in Figure 11a,b. The Bingham model was used for linear fitting [40] shown in Equation (6):

T = *G* + *HN* (6)

where T is torque (N·m), *N* is rotational speed (rev/s), *G* is the torque intercept (N·m), and *H* is the slope of the torque–rotational speed curve (N·m·s/rev).

Equation (6) describes the linear fits to the measured torque–rotational speed data. The dynamic yield stress and plastic viscosity were obtained using the ICAR software as described in Section 2.6. The coefficients of determination ranged from 0.9941 to 0.9990, showing that the measured torque–rotational speed data were well represented by the linear relation over the tested range [41,42]. With decreasing w/b, both G and H generally increased [43]. Across the two tests, H increased from 10.5830 to 15.7736 and from 10.4472 to 14.8262 N·m·s/rev for S1 mixtures, and from 10.6575 to 12.2116 and from 10.1851 to 12.9282 N·m·s/rev for S2 mixtures as w/b decreased from 0.17 to 0.15. The larger increase in H for the S1 group is consistent with the larger increase in mean plastic viscosity.

At w/b ratios of 0.17, 0.16, and 0.15, the mean dynamic yield stresses were 83.8, 124.9, and 157.2 Pa for the S1 group and 80.0, 100.7, and 133.8 Pa for the S2 group, respectively (Figure 11c). At w/b ratios of 0.17, 0.16, and 0.15, the dynamic yield stress of S1 was 4.75%, 24.03%, and 17.49% higher than that of S2, respectively. The S1 group had a higher dynamic yield stress at each tested w/b ratio. It should be noted that the relative difference in dynamic yield stress between the two sands was largest at a w/b ratio of 0.16 and did not increase continuously with decreasing w/b ratio, implying that the effect of w/b ratio on dynamic yield stress is not a simple monotonic amplification relationship.

Figure 11d shows that the plastic viscosity of UHPC prepared with both manufactured sands increased with decreasing w/b ratio, but the magnitudes of increase differed. At a w/b ratio of 0.17, the plastic viscosities of S1 and S2 were 208.0 Pa·s and 206.3 Pa·s, respectively, which were essentially equivalent. When the w/b ratio decreased to 0.16, the plastic viscosities of S1 and S2 increased to 235.3 Pa·s and 222.1 Pa·s, respectively, with S1 being 5.94% higher than S2. When the w/b ratio further decreased to 0.15, the corresponding values reached 301.3 Pa·s and 248.0 Pa·s, with S1 being 21.49% higher than S2. Compared with dynamic yield stress, plastic viscosity exhibited a more consistent widening of the relative difference between the two UHPC groups as the w/b ratio decreased. The rapid increase in plastic viscosity of the S1 group indicates that the mixture still needs to overcome greater continuous shear resistance after flow initiation, which also provides a rheological explanation for its significantly prolonged T500.

**Figure 12:**
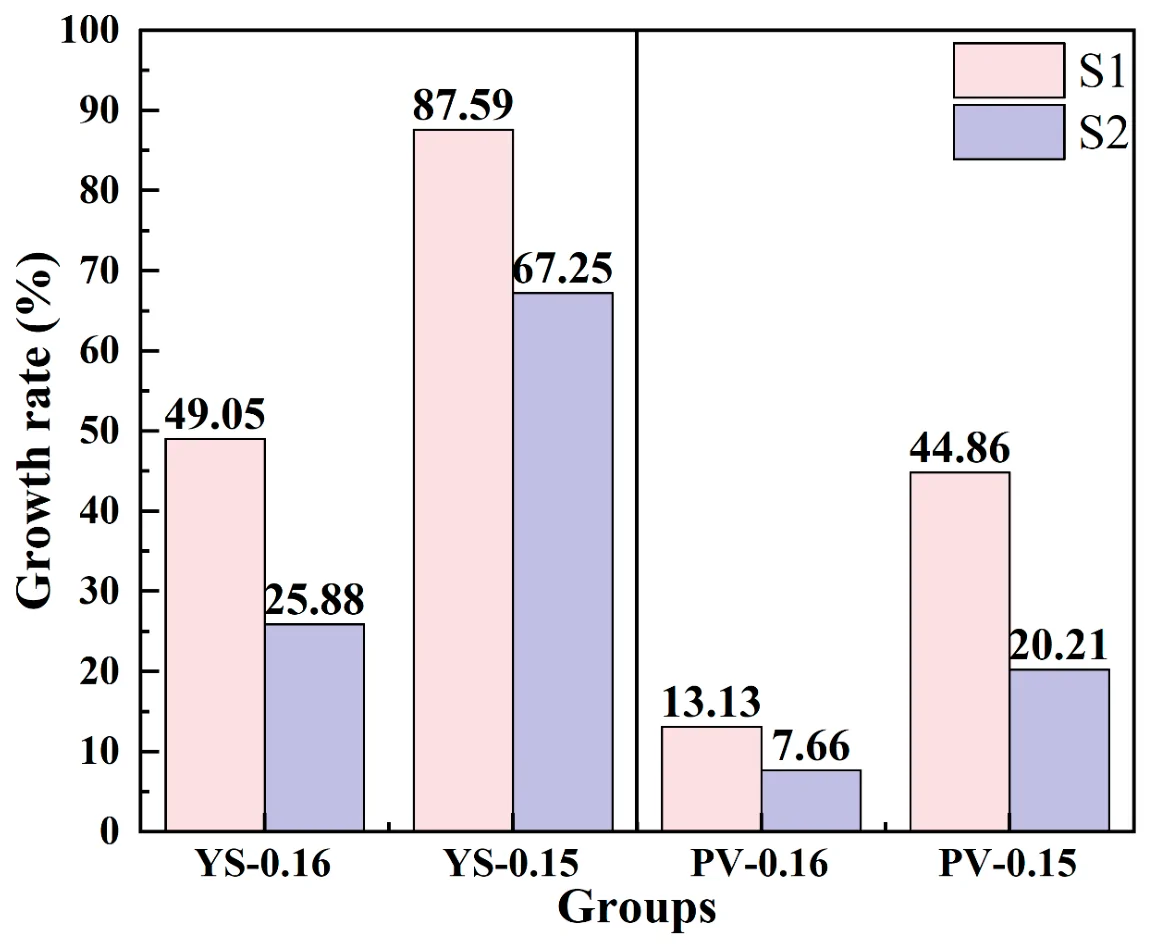
The growth rates of dynamic yield stress and plastic viscosity.

Relative increases were calculated using the value at w/b = 0.17 for each UHPC group as its reference. As shown in Figure 12, when the w/b ratio decreased to 0.16, the relative increases in dynamic yield stress of S1 and S2 were 49.05% and 25.88%, respectively; when the w/b ratio decreased to 0.15, the values increased to 87.59% and 67.25%, respectively. Dynamic yield stress increased more strongly in S1 than in S2 over the investigated w/b range. For plastic viscosity, the relative increases also differed between the two sand types: when the w/b ratio decreased to 0.16, S1 and S2 increased by 13.13% and 7.66%, respectively, and when the w/b ratio decreased to 0.15, S1 and S2 increased by 44.86% and 20.21%, respectively, with S1 being 24.64 percentage points higher than S2. The S1 group thus showed larger relative increases in both parameters. At matched w/b ratios, however, only the relative excess in plastic viscosity over S2 widened monotonically as w/b decreased.

Within the Bingham description, higher plastic viscosity indicates greater viscous resistance during established flow. Its widening relative difference between the two UHPC groups is consistent with the larger T500 contrast at lower w/b ratios. A possible explanation is that reduced particle lubrication makes continuous deformation more sensitive to source-related differences in particle interactions and dispersion. The relative difference in dynamic yield stress, however, peaked at w/b = 0.16, showing that the two parameters did not respond identically. These observations point to a particularly clear sand-source effect on continuous-flow resistance, while the underlying contributions remain to be resolved.

### 3.4. Mechanical Properties

**Figure 13:**
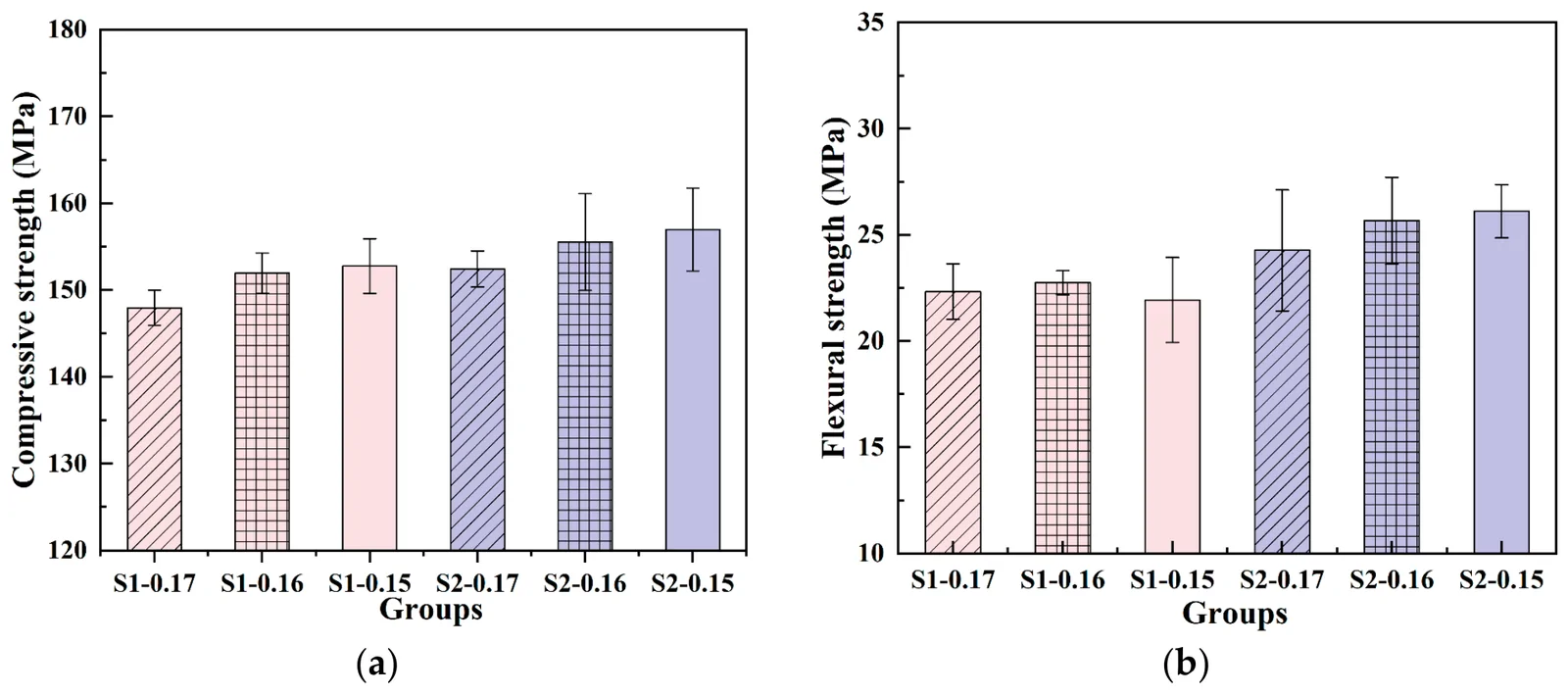
Mechanical properties of UHPC with different manufactured sands. (**a**) Compressive strength; (**b**) Flexural strength. Values are means of six cube specimens for compressive strength (*n* = 6 specimens per mixture) and three prismatic specimens for flexural strength (*n* = 3 specimens per mixture). Error bars represent the sample SD across the respective specimen results.

**Figure 14:**
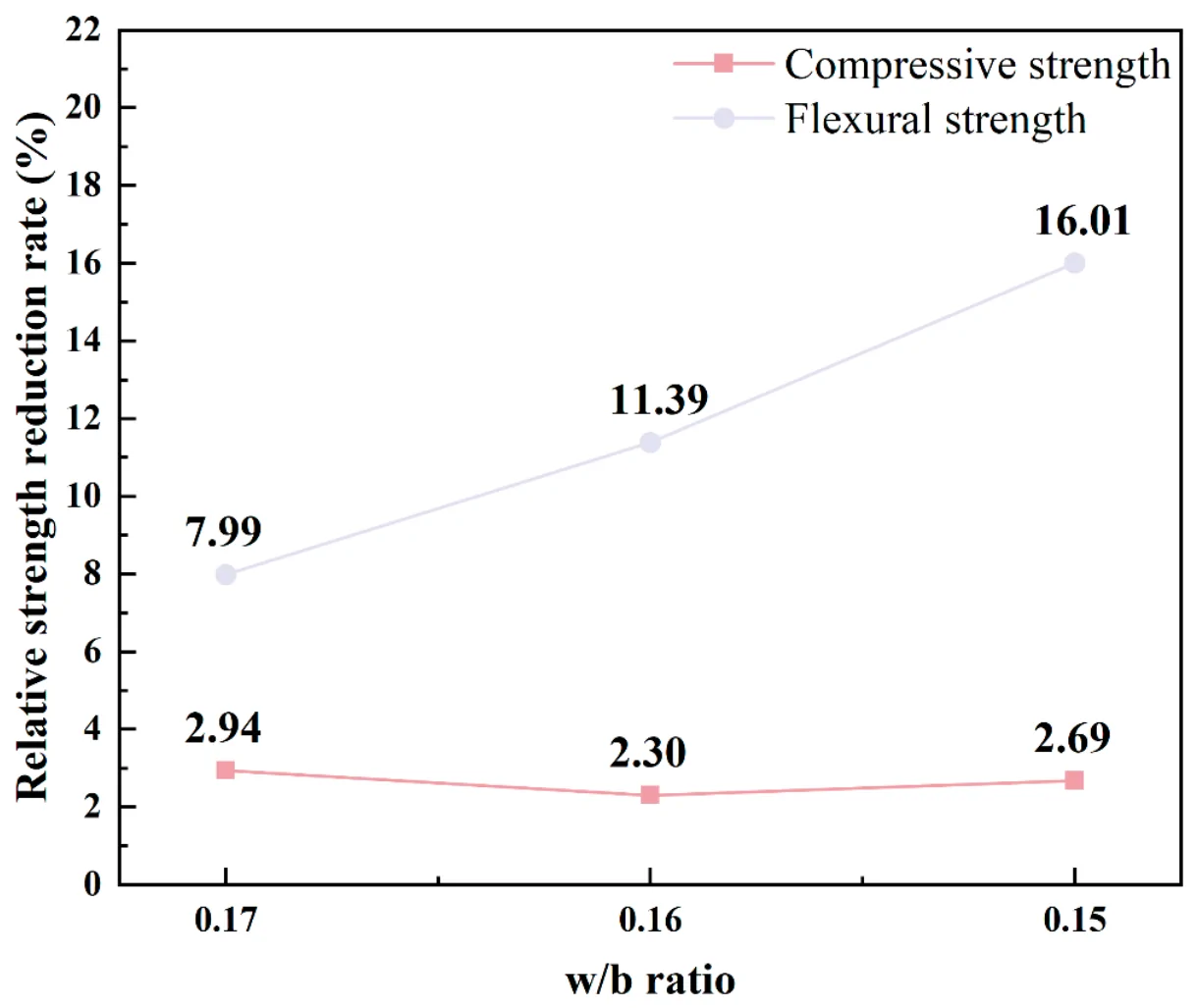
Reduction rate of strength.

Figure 13 and Figure 14 present the compressive and flexural strengths and the relative strength reductions in the S1 group compared with the S2 group. Compressive strength increased in both groups as the w/b ratio decreased from 0.17 to 0.15 (Figure 13a). The compressive strengths of the S1 group were 2.94%, 2.30%, and 2.69% lower than those of the S2 group at w/b ratios of 0.17, 0.16, and 0.15, respectively. The difference remained within approximately 2–3% and did not widen monotonically as w/b decreased.

As shown in Figure 13b, the flexural strength of UHPC prepared with the two manufactured sands exhibited different responses to the w/b. With decreasing w/b, the flexural strength of S2 gradually increased, whereas that of S1 increased slightly from 0.17 to 0.16 but decreased when the w/b was further reduced to 0.15. At the same w/b, the flexural strength of S1 was consistently lower than that of S2, with reduction rates of 7.99%, 11.39%, and 16.01%, respectively. Compared with compressive strength, the difference in flexural strength between the two sands was larger and continued to widen with decreasing w/b, indicating that the flexural performance of the S1 group is more sensitive to low w/b conditions.

As shown in Figure 14, the reduction rate of compressive strength remained at a relatively low level throughout, whereas the reduction rate of flexural strength gradually increased from 7.99% to 16.01%. This is because compressive strength is mainly governed by the compactness of the cementitious matrix and the load-bearing capacity of hydration products, and a lower w/b is generally beneficial for reducing capillary pores and enhancing matrix strength. In contrast, the flexural performance of steel fiber-reinforced UHPC is influenced not only by matrix strength but also by defect distribution, fiber dispersion and orientation, fiber–matrix interfacial bonding, and crack bridging, and is therefore more sensitive to the forming state of the fresh mixture.

Combined with the results in Section 3.2 and Section 3.3, S1 exhibited lower flow spread, longer T500, and higher plastic viscosity at low w/b. These results are consistent in trend, indicating that excessively high continuous flow resistance may be unfavorable to air expulsion, elimination of defects, and uniform distribution of steel fibers during casting, thereby weakening the bridging effect of fibers on flexural cracks. From the gradation-weighted results in Section 3.1, S1 exhibited a relatively stronger tendency for PCE adsorption, which may reduce the effective PCE available in the liquid phase for dispersing cementitious particles. Therefore, the difference in PCE adsorption can provide a material-level explanation for the differences in rheological and mechanical responses between UHPC prepared with the two manufactured sands.

### 3.5. Cross-Sectional Pore Distribution

**Figure 15:**
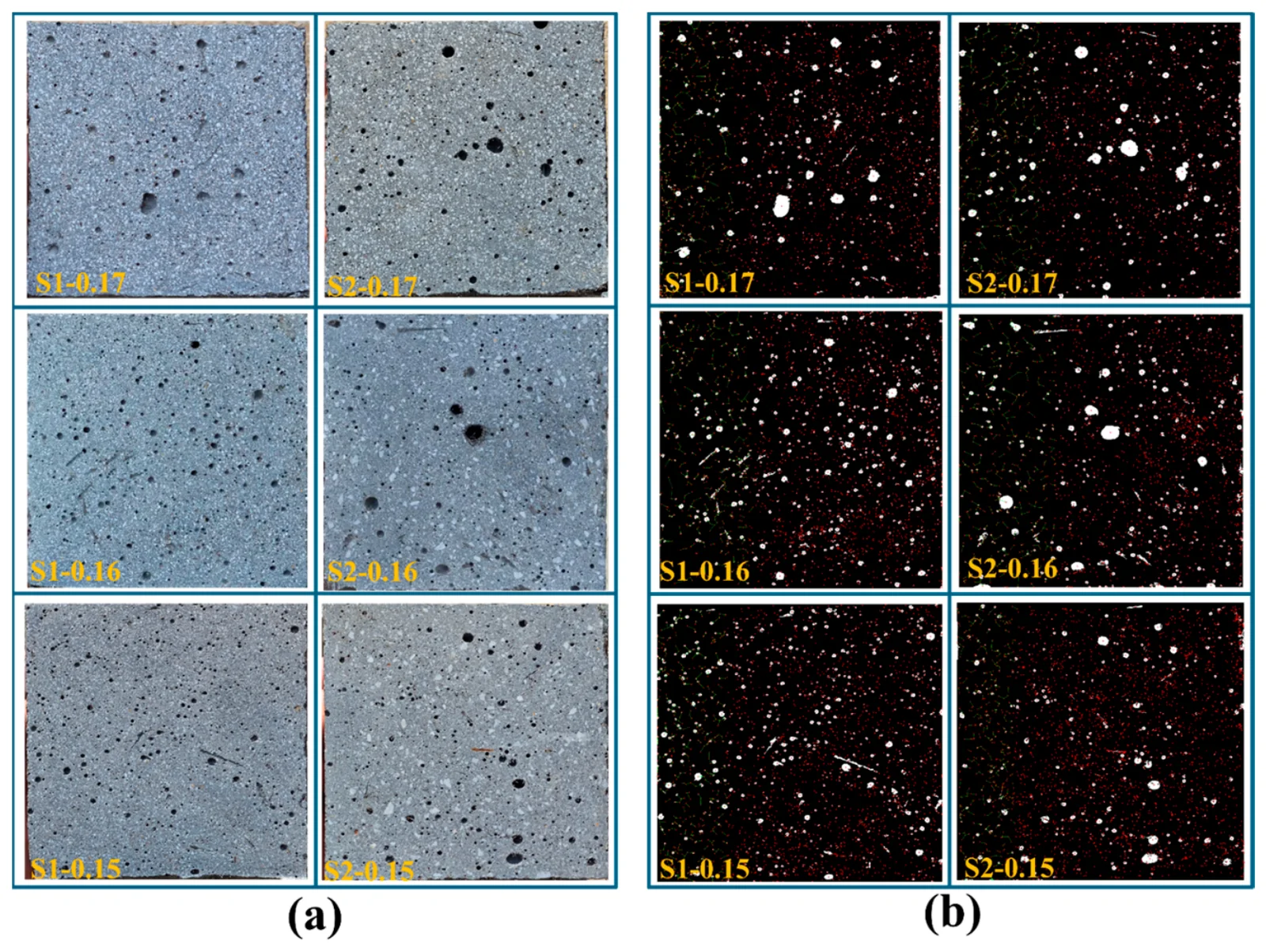
Pore diagram of sample interface. (**a**) Sample cross-section; (**b**) Binarization and pore identification.

**Figure 16:**
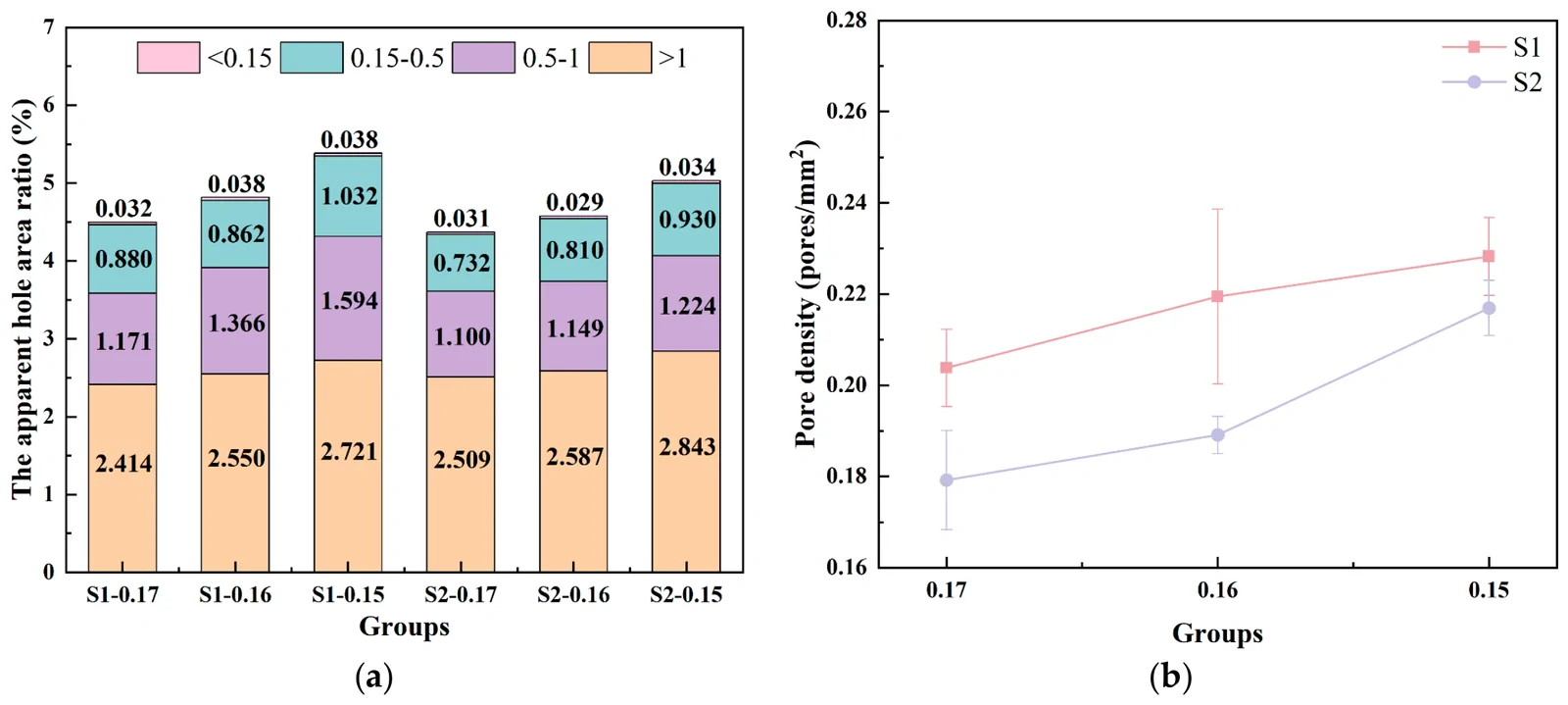
Porosity distribution characteristics. (**a**) Visible pore area ratio; (**b**) Pore density. Values are means of measurements on two opposite end sections of one prismatic specimen (*n* = 2 sections; one specimen). Error bars in (**b**) represent the sample SD between the two section values and describe within-specimen spatial variation. The two sections are spatial observations within a single specimen.

Figure 15 presents the original images and pore identification results for one end section of each UHPC prism; the corresponding images for the opposite end are provided in Appendix A. For each mixture, pore indicators were measured on the two opposite end sections of a single prism and averaged for the comparison in Figure 16. The two sections are not independent experimental replicates. These sections represent two spatial observation areas within the same specimen. Examining both ends extends the observation beyond a single section and reduces reliance on a single cross-section. Consistent image acquisition and processing provide a common basis for comparing visible pore area fraction, number density, and size distribution among the examined specimens [44,45,46].

Figure 16a shows that the mean total visible pore area fraction increased in both UHPC groups as w/b decreased from 0.17 to 0.15. The total area fractions of S1 at the three w/b ratios were approximately 4.497%, 4.816%, and 5.385%, respectively, while those of S2 were approximately 4.372%, 4.575%, and 5.031%, respectively. Based on the previous experimental results, S1 exhibited a stronger tendency for adsorption or solid-phase retention of PCE-related organic components. Under the condition of the same PCE dosage, this may reduce the effective PCE available in the liquid phase for dispersing cementitious particles such as cement and silica fume. At a relatively high w/b, the system still possesses a certain free water and dispersion margin, so the influence of this adsorption difference on the forming state is relatively limited. However, as the w/b decreases, the interparticle distance and the thickness of the surface water film decrease, and the dual margins of free water and effective PCE narrow simultaneously. Under such conditions, even a small adsorption difference is more easily converted into significant differences in particle dispersion state and rheological resistance. Therefore, the gradually widening gap in visible pore area fraction between S1 and S2 reflect the amplified sensitivity effect of the PCE adsorption difference under low w/b conditions.

In terms of pore size composition, pores larger than 1 mm were the main contributors to the cross-sectional pore area in all groups, but at the same w/b, the area fraction of S1 in this range was slightly lower than that of S2. The higher total pore area fraction of S1 mainly originated from pores in the range of 0.15–1 mm, especially the increased contribution from pores of 0.5–1 mm. Combined with the aforementioned workability and rheological results, S1 exhibited a longer T500 and higher plastic viscosity at lower w/b, indicating that its continuous flow and local rearrangement were subjected to greater resistance. Under the non-vibrated casting condition adopted in this study, higher viscous resistance may restrict the migration, coalescence, and escape of air bubbles with paste deformation, causing some bubbles to remain in the hardened matrix as medium-sized pores and thereby increasing the visible pore area fraction in the cross-section. This also suggests that, compared with the final flow spread, the plastic viscosity and T500, which reflect continuous flow resistance, may be more directly related to the pore state formed in hardened UHPC.

Figure 16b shows that the mean visible pore number density calculated from the two end sections increased with decreasing w/b ratio in both UHPC systems. S1 generally exhibited a higher pore number density than S2. Meanwhile, the increasing difference in total visible pore area fraction suggests that the pore characteristics differed in both number and size distribution. Huang et al. [47] reported that lower dynamic yield stress or plastic viscosity was associated with reduced air-bubble content in hardened UHPC. Although their study used higher w/b ratios of 0.21–0.27, this relationship is qualitatively consistent with the higher plastic viscosity and visible pore area fraction observed for S1 at w/b ratios of 0.16 and 0.15 in the present study. Under the non-vibrated casting conditions used here, greater viscous resistance may have hindered air-bubble migration and escape, contributing to the higher visible pore area fraction. The higher apparent PCE adsorption index of S1 may have contributed to this behavior through reduced particle dispersion and increased flow resistance.

## 4. Discussion

### 4.1. Multi-Indicator Relative Response Analysis

**Figure 17:**
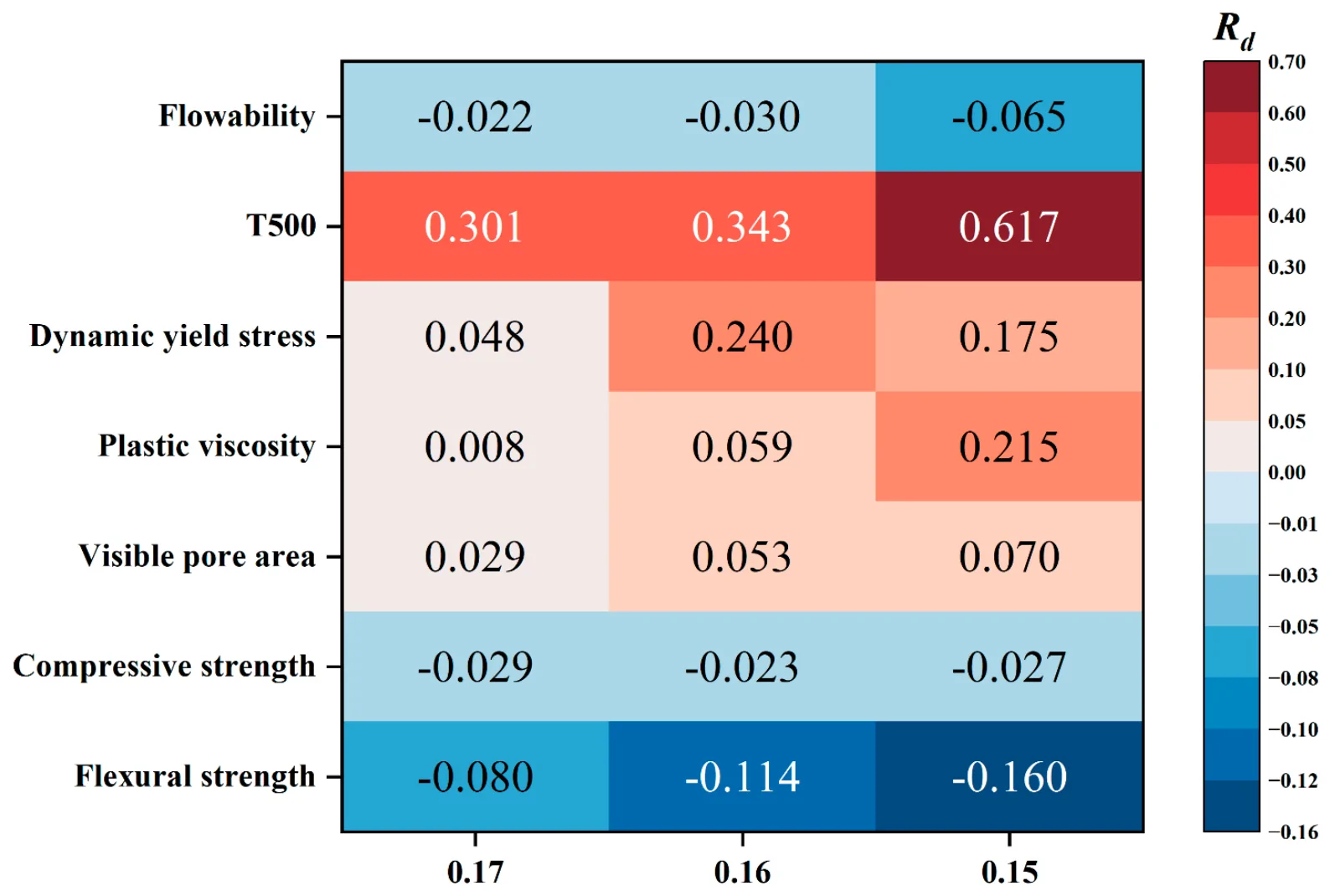
Relative differences in the measured UHPC properties, with the S2 group used as the reference at each w/b ratio.

Figure 17 compares the relative differences between the two UHPC groups across the measured properties. The relative differences are descriptive comparisons based on the reported mean values. The fresh-state measurements characterize variation between two samples from a single batch per mixture, whereas the pore measurements characterize spatial variation between two end sections of one specimen. These observations provide a basis for comparing the tested mixtures and examined sections. For each w/b ratio, the S2 group was used as the reference, and the relative difference, Rd, was calculated using Equation (7):

(7) $$R_{d}=\frac{X_{S1}-X_{S2}}{X_{S2}}$$

where *X_S_*_1_ and *X_S_*_2_ denote the measured values for the S1 and S2 groups at the same w/b ratio. A positive *R_d_* indicates a higher measured value for the S1 group.

As shown in Figure 17, under the three w/b ratios, S1 consistently exhibited a smaller flow spread, a longer T500, a higher dynamic yield stress, and a higher visible pore area fraction. Meanwhile, its compressive and flexural strengths were lower than those of S2. The response of plastic viscosity was strongly dependent on the w/b ratio: when the w/b ratio was 0.17, the mean plastic viscosities of the S1 and S2 groups were similar, with *R_d_* being only 0.008; when the w/b ratio decreased to 0.16 and 0.15, *R_d_* increased to 0.059 and 0.215, respectively. These results show that UHPC mixtures containing the two manufactured sands differ in fresh-state flow spread, fresh rheological properties, cross-sectional visible pore area fraction, and the compressive and flexural strengths of hardened specimens.

The *R_d_* of the visible pore area ratio increased from 0.029 to 0.070 as the w/b ratio decreased, while the *R_d_* of the flexural strength decreased from −0.080 to −0.160. The widening relative difference in visible pore area fraction was accompanied by an increasing flexural strength deficit. This suggests that, under low free-water conditions, variations in fresh-state flow and casting may be associated with differences in cross-sectional visible pore characteristics and flexural performance after hardening. The inter-group relative difference in dynamic yield stress peaked at w/b = 0.16, whereas the compressive strength deficit remained within approximately 2–3%. Neither followed the monotonic widening observed for plastic viscosity. This suggests that the sand source difference does not passively transfer to all performance indicators. This can be explained by the fact that the compressive strength is also controlled by factors such as the hydration degree of the matrix and the multi-scale pore structure, and that the matrix densification effect produced by reducing the w/b ratio may, to a certain extent, weaken the influence of the difference in visible pores on the compressive strength. In contrast, the flexural performance is more sensitive to the cross-sectional defect and crack propagation conditions. In summary, as the w/b ratio decreases, the free water and dispersion margin narrow, the differences in some indicators between S1 and S2 expand overall, while a few indicators remain relatively stable. This indicates that the indicator-selective amplification characteristic of the sand source-PCE compatibility difference is manifested under low w/b ratios.

### 4.2. Discussion of Proposed Mechanisms

**Figure 18:**
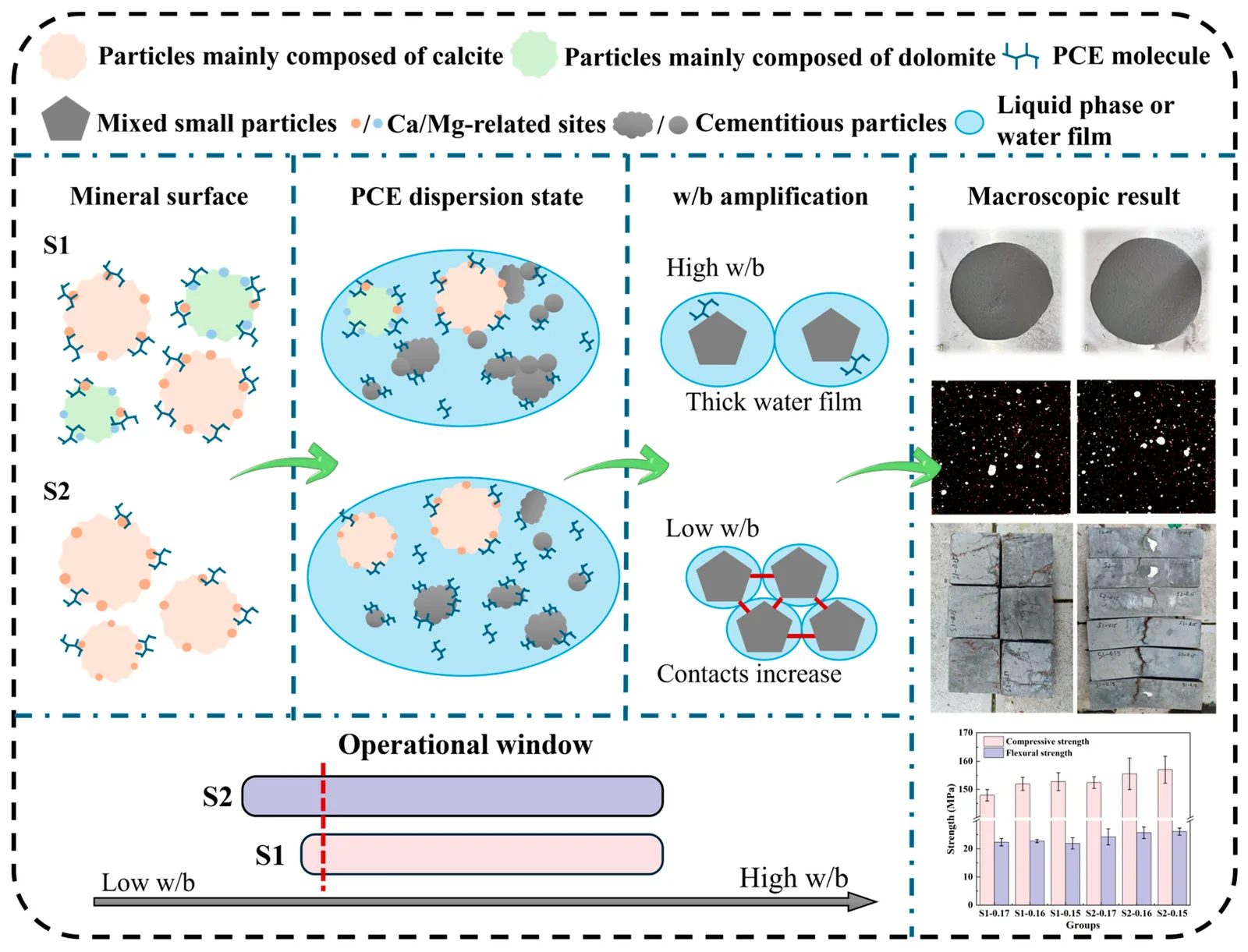
Proposed mechanistic interpretation of sand–PCE compatibility and its possible implications for UHPC performance at low w/b ratios.

Based on the above results, the performance differences between UHPC prepared with the two manufactured sands may be attributed to the combined effects of the mineral composition and surface characteristics of the manufactured sand and the low w/b ratio condition. The Rietveld-refined XRD data and chemical composition analysis indicated that S1 contained a distinct dolomite component, with a significantly higher MgO content than S2, which was dominated by calcite. Meanwhile, the stone powder contents and methylene blue values of the two manufactured sands were similar, and the methylene blue value of S1 was not higher than that of S2. Therefore, mineral composition, together with potential differences in particle morphology and surface condition, may contribute to the higher apparent PCE adsorption index of S1. Figure 18 presents a proposed mechanistic interpretation of the observed performance differences. The depicted PCE distribution and particle configurations are conceptual and were not directly verified. The compatibility ranges shown on the w/b axis are illustrative and do not represent experimentally established thresholds.

The chemical composition of dolomite is CaMg(CO_3_)_2_, and the ordered distribution of Ca and Mg sites in its crystal structure differs from that of calcite [24]. The fresh fracture surfaces, structural defects, and surface hydroxylation state formed during the crushing process of manufactured sand may further alter the Ca/Mg-related sites, local surface charge, and interfacial ionic environment of the particles, thereby affecting the electrostatic interaction, ionic coordination, and bridging behavior between the carboxyl groups of PCE and the carbonate particle surfaces. The dolomite component and corresponding surface characteristics of S1 are consistent with its stronger tendency for adsorption or solid-phase retention of PCE-related organic components. In a multi-component UHPC system, the added PCE may be distributed among manufactured sand particles, cementitious binder particles, and the liquid phase [48]. For conceptual discussion, this distribution can be represented by a simplified partitioning balance:

*D_PCE_* = *D*_sand_ + *D*_binder_ + *D*_liquid_ (8)

where *D_PCE_* denotes the total added PCE dosage, and *D_sand_*, *D_binder_*, and *D_liquid_* denote the PCE dosage contributions associated with manufactured sand particles, cementitious binder particles, and the liquid phase, respectively. All terms are expressed on the same PCE mass basis per unit volume of UHPC. Equation (8) is a conceptual representation; the individual contributions were not quantified in the UHPC mixtures. PCE adsorption on binder particles can promote dispersion through steric hindrance. If the difference in apparent PCE retention observed in the sand–solution tests also occurs in UHPC, greater sand-associated retention could reduce PCE availability for dispersing binder particles at a fixed total dosage.

This difference may not induce significant macroscopic responses at relatively high w/b, because the system still possesses a certain amount of free water and dispersion margin. However, as the w/b decreases, the solid volume fraction increases, the water film thickness decreases, and the interparticle distance is shortened, which will consequently enhance particle contact and friction [37,49]. Previous studies have indicated that the rheological behavior of low w/b systems is controlled by both the particle contact network and the degree of PCE surface coverage; when the system approaches a critical dispersion state, even a small change in the effective PCE content may produce a nonlinear rheological response [50]. The relative differences shown in Figure 17 further support this selective amplification characteristic. When the w/b decreased from 0.17 to 0.15, the increase in T500 of S1 relative to S2 expanded from 30.1% to 61.7%, the relative difference in plastic viscosity increased from 0.8% to 21.5%, and the difference in flow spread also increased from −2.2% to −6.5%. The relative difference in dynamic yield stress increased from 4.8% at w/b = 0.17 to 24.0% at w/b = 0.16, before decreasing to 17.5% at w/b = 0.15. Plastic viscosity therefore showed a more consistent widening of the inter-group difference with decreasing w/b ratio, highlighting the contrast in continuous-flow resistance. For UHPC without external vibration, the continuous flow capacity not only determines the spreading process of the paste but also affects the migration and escape of air bubbles during casting [47]. When the effective PCE dispersion margin of the S1 group decreases and the plastic viscosity and dynamic flow resistance increase, the upward migration and expulsion of air bubbles in the paste may be restricted. Cross-sectional analysis (Figure 16) showed that the higher total visible pore area fraction of S1 mainly originated from the area contribution of pores in the range of 0.15–1 mm, whereas its area fraction of pores not smaller than 1 mm was not higher than that of S2. Therefore, the pore difference between the two sands is more likely manifested as an increase in the retention of small- and medium-scale visible pores. The influence of this pore characteristic on mechanical properties can be referred to in the discussion presented in Section 3.5.

These considerations motivate a conceptual framework for PCE dosage selection:

*D*_min_(*w*/*b*,sand) ≤ *D*_PCE_ ≤ *D*_max_(*w*/*b*,sand) (9)

The proposed effective PCE dosage window extends from *D_min_*, the minimum dosage required to achieve the target flow spread and fresh rheological properties, to *D_max_*, the upper limit compatible with acceptable mixture stability and setting behavior. Differences in the mineral composition and surface characteristics of manufactured sands may influence apparent PCE adsorption and its availability for dispersing cementitious particles, suggesting that this dosage window may be source-dependent. For the calcite–dolomite sand, greater apparent PCE adsorption may increase the dosage required for adequate dispersion, potentially shifting *D_min_* toward a higher value. This potential dependence on sand source may become more relevant at low w/b ratios, where the water and dispersion margins are reduced. The proposed framework therefore highlights the importance of considering sand–PCE compatibility at the intended w/b ratio when selecting the PCE dosage [50].

The higher apparent PCE adsorption index of S1 may reflect the combined effects of mineral composition and other sand-source-related particle characteristics. Although the two sands were adjusted to the same gradation, the effects of particle morphology and surface condition were not isolated. Their individual contributions to the observed differences cannot be determined from the present data. Further research should characterize these factors to distinguish their contributions from those of mineral composition. The present study provides an initial assessment of compatibility differences that may arise when using carbonate manufactured sands from different engineering sources in UHPC.

## 5. Conclusions

This study investigated the effects of a calcite–dolomite manufactured sand (S1) and a calcite-dominated manufactured sand (S2) on apparent PCE adsorption, flowability behavior, fresh rheological properties, cross-sectional visible porosity, and mechanical properties of ultra-high performance concrete (UHPC) at water-to-binder (w/b) ratios ranging from 0.17 to 0.15. The main conclusions are as follows:

(1) The calcite–dolomite manufactured sand exhibited a consistently stronger tendency for adsorption or solid-phase retention of PCE-related organic components. Across all four size fractions, the apparent PCE adsorption index of the calcite–dolomite manufactured sand was consistently higher, and its gradation-weighted value was 2.23% higher than that of the calcite-dominated manufactured sand.
(2) As the w/b ratio decreased, the differences in fresh-state behavior between the two systems were selectively amplified. At a w/b ratio of 0.15, the calcite–dolomite manufactured sand system exhibited a 6.5% lower flow spread, a 61.7% longer T500, a 17.5% higher dynamic yield stress, and a 21.5% higher plastic viscosity compared with the calcite-dominated system. As the w/b ratio decreased from 0.17 to 0.15, dynamic yield stress increased by 87.6% in S1 and 67.3% in S2, while plastic viscosity increased by 44.9% and 20.2%, respectively, showing larger relative increases in both parameters for S1. These trends may reflect the combined influence of sand–PCE compatibility, reduced water availability, and increased particle interactions on dispersion and viscous resistance at low w/b ratios.
(3) The average visible pore area fraction increased with decreasing w/b, reaching 5.385% and 5.031% for the calcite–dolomite manufactured sand system and the calcite-dominated system, respectively, at a w/b ratio of 0.15. At matched w/b ratios, UHPC containing calcite–dolomite sand exhibited approximately 2–3% lower compressive strength than UHPC containing calcite-dominated sand, whereas its flexural strength deficit widened from 8.0% to 16.0% as w/b decreased.
(4) A source-dependent effective PCE dosage window is proposed as a conceptual framework for examining sand–PCE compatibility at low w/b ratios. The framework emphasizes considering sand–PCE compatibility at the intended w/b ratio when selecting a PCE dosage. Its lower and upper boundaries were not determined in this study and require validation through tests at multiple PCE dosages.

## Figures and Tables

**Table 1:** Chemical Composition of Raw Materials (wt.%).

	CaO	SiO_2_	Al_2_O_3_	Fe_2_O_3_	MgO	Na_2_O	K_2_O	SO_3_	TiO_2_	P_2_O_5_
S1	93.03	0.29	0.22	0.23	6.08	0.06	0.01	0.02	—	0.01
S2	98.10	0.35	0.17	0.28	0.83	—	0.01	0.03	—	0.02
OPC	66.47	16.55	4.34	4.39	2.03	0.20	1.04	3.64	0.64	0.15
SF	0.85	95.12	0.31	0.09	0.67	0.17	0.63	0.21	—	0.20
FA	14.19	41.53	18.88	6.33	3.79	2.36	2.28	3.91	1.20	1.06

Note: The XRF results for S1 and S2 are reported as oxide-equivalent compositions normalized to 100% over the measured components. CO_2_ and LOI were excluded from the normalization sum. These values describe the normalized composition of the reported components and should not be interpreted as independently verified whole-rock oxide mass fractions.

**Table 2:** Physical Properties of Sand.

	Loose Bulk Density (kg/m^3^)	Packed Bulk Density (kg/m^3^)	Apparent Density (kg/m^3^)	Porosity (%)	Crushing Value (%)	Stone Powder (%)	Methylene Blue
S1	1481	1658	2694	38.5	16.2%	6.9	0.25
S2	1452	1632	2733	40.3	14.2%	7.3	0.50

**Table 3:** Mix Proportions of UHPC (kg/m^3^).

	OPC	SF	FA	Sand	Fiber	PCE	Water
S1-0.17	800	180	170	1050	195	24	183
S1-0.16	800	180	170	1050	195	24	172
S1-0.15	800	180	170	1050	195	24	160
S2-0.17	800	180	170	1050	195	24	183
S2-0.16	800	180	170	1050	195	24	172
S2-0.15	800	180	170	1050	195	24	160

**Figure A1:**
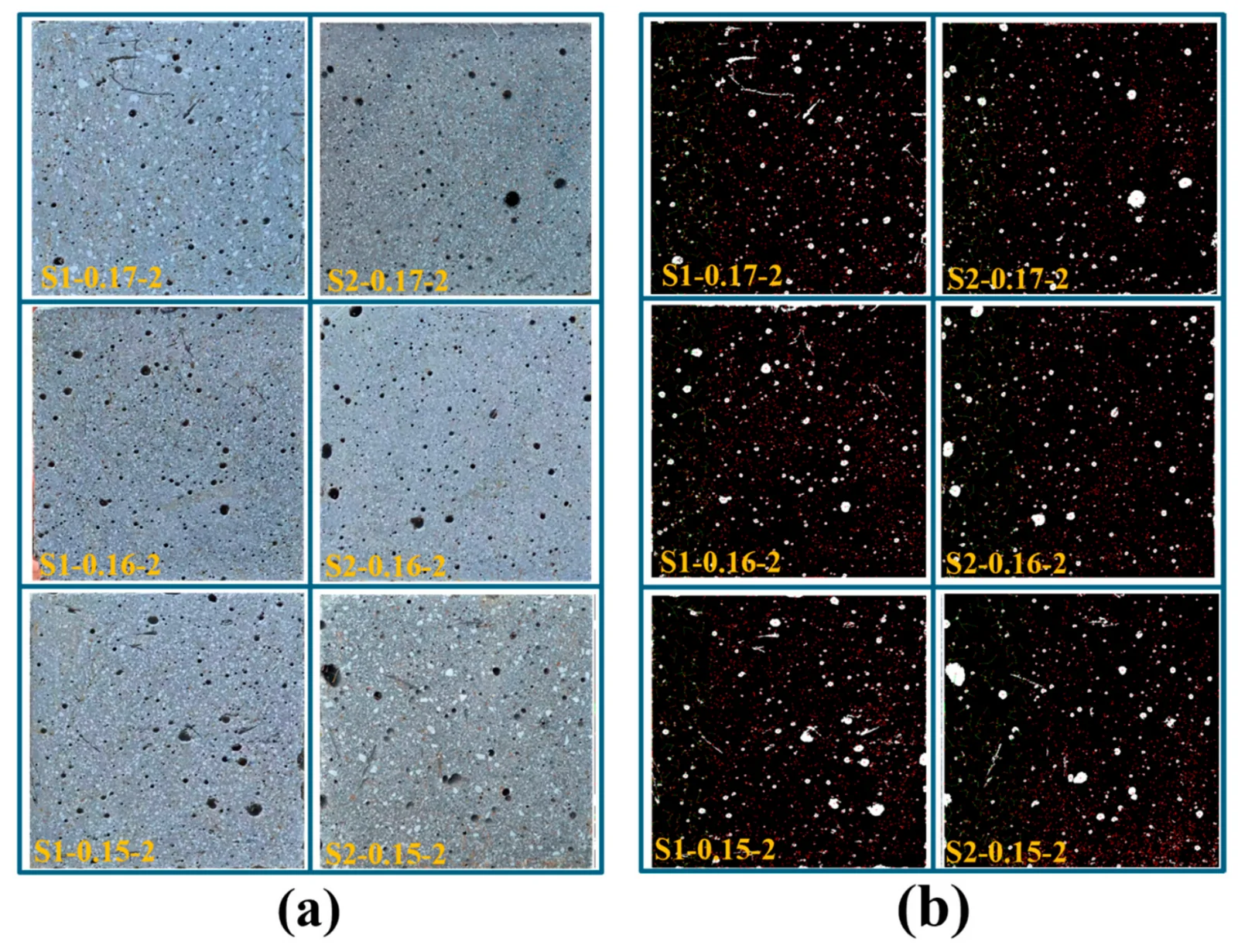
S1 and S2 Right end section and identification diagram. (**a**) Sample cross-section; (**b**) Binarization and pore identification.

## Data Availability

The raw data supporting the conclusions of this article will be made available by the authors on request.

## Appendix A

Figure A1 shows the original and binary images of the opposite (right) end sections of the S1 and S2 UHPC specimens, complementing the left-end images in Figure 15. For each mixture, the two end sections were taken from one specimen and represent spatial observation areas rather than independent experimental replicates.

The cross-sectional images were acquired under high-contrast illumination. In the original images, the pores appear as dark regions against the gray cementitious matrix. The images were subsequently converted to grayscale and subjected to smoothing, filtering, and threshold segmentation in Fiji. The resulting binary images were analyzed in MATLAB using an 8-connected-component algorithm. The projected area and centroid of each identified pore were determined, and its equivalent diameter was calculated from the actual projected area. Pore area fraction, pore number density, and area fractions within different equivalent-diameter ranges were then obtained for each section.
